# Glycan-specific IgM is critical for human immunity to *Staphylococcus aureus*

**DOI:** 10.1016/j.xcrm.2024.101734

**Published:** 2024-09-17

**Authors:** Astrid Hendriks, Priscilla F. Kerkman, Meri R.J. Varkila, Jelle L.G. Haitsma Mulier, Sara Ali, Thijs ten Doesschate, Thomas W. van der Vaart, Carla J.C. de Haas, Piet C. Aerts, Olaf L. Cremer, Marc J.M. Bonten, Victor Nizet, George Y. Liu, Jeroen D.C. Codée, Suzan H.M. Rooijakkers, Jos A.G. van Strijp, Nina M. van Sorge

**Affiliations:** 1 Department of Medical Microbiology and Infection Prevention, Amsterdam UMC, location University of Amsterdam, Amsterdam, the Netherlands; 2 Department of Medical Microbiology, University Medical Center Utrecht, Utrecht University, Utrecht, the Netherlands; 3 Julius Center for Health Sciences and Primary Care, University Medical Center Utrecht, Utrecht University, Utrecht, the Netherlands; 4 Department of Intensive Care Medicine, University Medical Center Utrecht, Utrecht University, Utrecht, the Netherlands; 5 Leiden Institute of Chemistry, Leiden University, Leiden, the Netherlands; 6 Department of Internal Medicine, Division of Infectious Diseases, Amsterdam University Medical Center, University of Amsterdam, Amsterdam, the Netherlands; 7 Department of Pediatrics, University of California San Diego, San Diego, CA, USA; 8 Netherlands Reference Center for Bacterial Meningitis, Amsterdam UMC, location AMC, Amsterdam, the Netherlands; 9 Amsterdam Institute for Infection and Immunity, Infectious Diseases, Amsterdam, The Netherlands

**Keywords:** *Staphylococcus aureus*, glycan, wall teichoic acid, WTA, antibody, IgM, protective immunity, bacteremia, opsonic, protein A

## Abstract

*Staphylococcus aureus* is a major human pathogen, yet the immune factors that protect against infection remain elusive. High titers of opsonic IgG antibodies, achieved in preclinical animal immunization studies, have consistently failed to provide protection in humans. Here, we investigate antibody responses to the conserved *S. aureus* surface glycan wall teichoic acid (WTA) and detect the presence of WTA-specific IgM and IgG antibodies in the plasma of healthy individuals. Functionally, WTA-specific IgM outperforms IgG in opsonophagocytic killing of *S. aureus* and protects against disseminated *S. aureus* bacteremia through passive immunization. In a clinical setting, patients with *S. aureus* bacteremia have significantly lower WTA-specific IgM but similar IgG levels compared to healthy controls. Importantly, low WTA-IgM levels correlate with disease mortality and impaired bacterial opsonization. Our findings may guide risk stratification of hospitalized patients and inform future design of antibody-based therapies and vaccines against serious *S. aureus* infection.

## Highlights

• *S. aureus* WTA-specific IgM and IgG antibodies are present in healthy individuals
• WTA-specific IgM outperforms IgG in bacterial opsonization and neutrophil killing
• Opsonic IgM effector functions are not hindered by the major virulence factor SpA
• Association between low WTA-IgM and mortality in patients with *S. aureus* bacteremia

## Graphical abstract

**Figure:**
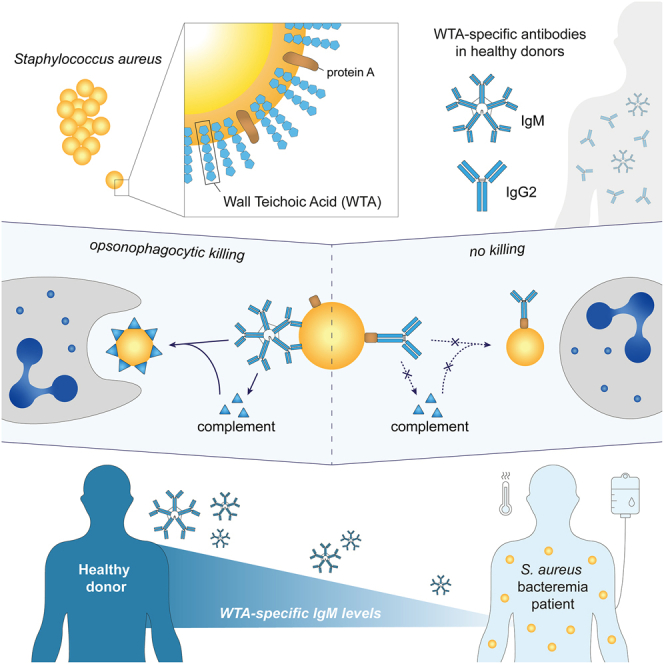

## Teaser

It is currently unknown which immune factors protect against *Staphylococcus aureus* infection. Hendriks et al. report an important role for opsonic IgM antibodies in defense against severe *S. aureus* infection. This study has implications for clinical care management, the development of immune-based therapies, and vaccine design.

## Introduction

The bacterial pathogen *Staphylococcus aureus* is among the leading causes of both community- and hospital-acquired infections in human medicine. Despite significant advances in intensive care management, high rates of morbidity and mortality persist for patients with *S. aureus* bacteremia.^1^ This problem is further compounded by the emergence of antibiotic-resistant strains, particularly methicillin-resistant *S. aureus* (MRSA), which hinders both treatment and prophylaxis efforts.^2^ To improve future clinical outcomes, improved risk stratification as well as the development of alternative strategies, such as vaccines and immune-based approaches, is imperative. However, a precise understanding of the immune correlates responsible for protection against *S. aureus* remains elusive.

Neutrophils play a critical role in eradicating *S. aureus*, as demonstrated by human genetic studies and animal experiments showing that defects in neutrophil responses predispose individuals to infection.^3^^,^^4^ To effectively combat *S. aureus*, neutrophils rely on additional immune factors, such as opsonic antibodies and complement. Ig-mediated complement activation leads to the deposition of C3b, enhancing bacterial uptake and killing by neutrophils, and promotes neutrophil recruitment through the release of potent chemotactic factors such as C5a.^5^^,^^6^ Despite the success of opsonic IgG antibodies against other bacterial pathogens, such as pneumococci, meningococci, and *Haemophilus influenzae* type b (Hib), efforts to identify phagocytosis-enhancing IgG targeting conserved surface structures for *S. aureus* have faced challenges.^7^^,^^8^^,^^9^ Therapeutic antibodies and vaccines targeting surface-exposed antigens that showed promise in preclinical animal models have consistently failed in human clinical trials, highlighting the need for antibody profiling studies in relevant patient cohorts to improve predictive value.^7^^,^^8^

One of the most prominent *S. aureus* virulence factors is staphylococcal protein A (SpA), an abundant and universally expressed surface molecule.^10^^,^^11^ SpA effectively inhibits Fc-mediated IgG effector functions, including complement activation and Ig-mediated phagocytosis.^12^^,^^13^ SpA-mediated IgG interference may explain the limited effectiveness of IgG against *S. aureus* in real-world studies. However, IgG is not the only antibody isotype capable of boosting neutrophil activity. IgM, the third most abundant isotype in blood, exhibits strong complement activation capacity.^14^ Importantly, SpA does not bind the Fc region of IgM,^15^ suggesting that its opsonic capacity remains unaffected by SpA. Furthermore, IgM shows enhanced binding to structures with repetitive epitopes, such as microbial carbohydrates, due to its higher avidity compared to other Ig isotypes.^16^ Despite these potential benefits, the role of *S. aureus*-specific IgM in host defense has received limited research attention.

*S. aureus* wall teichoic acids (WTAs) are conserved cell-wall-anchored glycopolymers and a dominant target for opsonic antibodies, with as much as 70% of the total surface-directed IgG pool binding to WTA.^17^ *S. aureus* WTAs exhibit distinct structural variation due to glycosylation with *N*-acetylglucosamine (GlcNAc), impacting immune interactions and antibody recognition.^18^^,^^19^ Three WTA glycotypes have been identified, each requiring a dedicated glycosyltransferase enzyme (TarM, TarS, or TarP) to add the GlcNAc to the ribitol phosphate (RboP) WTA backbone subunits in an α1,4 (TarM), β1,4 (TarS), or β1,3 (TarP) position.^20^^,^^21^^,^^22^ All *S. aureus* isolates possess the *tarS* gene, while approximately 40% of strains co-contain *tarM* or *tarP*.^23^ The specific WTA glycoprofile of a particular *S. aureus* strain is determined by the presence of corresponding *tar* genes, enzymatic activity, and environmental conditions, leading to glycovariation.^24^ The diversity in *S. aureus* WTAs holds important implications for immune responses and potential antibody-based therapeutics.

IgG antibodies, predominantly IgG2, have been detected against all three WTA-GlcNAc glycotypes in serum from healthy donors, with a higher reactivity toward β1,4-GlcNAc and β1,3-GlcNAc compared to α1,4-GlcNAc-WTA.^18^ These antibodies enhanced complement deposition and neutrophil killing of SpA-deficient *S. aureus*.^25^^,^^26^^,^^27^ Despite the widespread reactivity of WTA-reactive antibodies in healthy individuals, their potential contribution to protection against severe *S. aureus* infections in humans remains unexplored. In this study, we used synthetic WTA oligomers that mimic *S. aureus* WTA to investigate human systemic antibody responses to the three main *S. aureus* WTA glycotypes. Unexpectedly, our studies revealed a critical role for WTA-specific IgM in immune protection.

## Results

### The antibody repertoire to *S. aureus* WTA in healthy individuals

**Figure 1:**
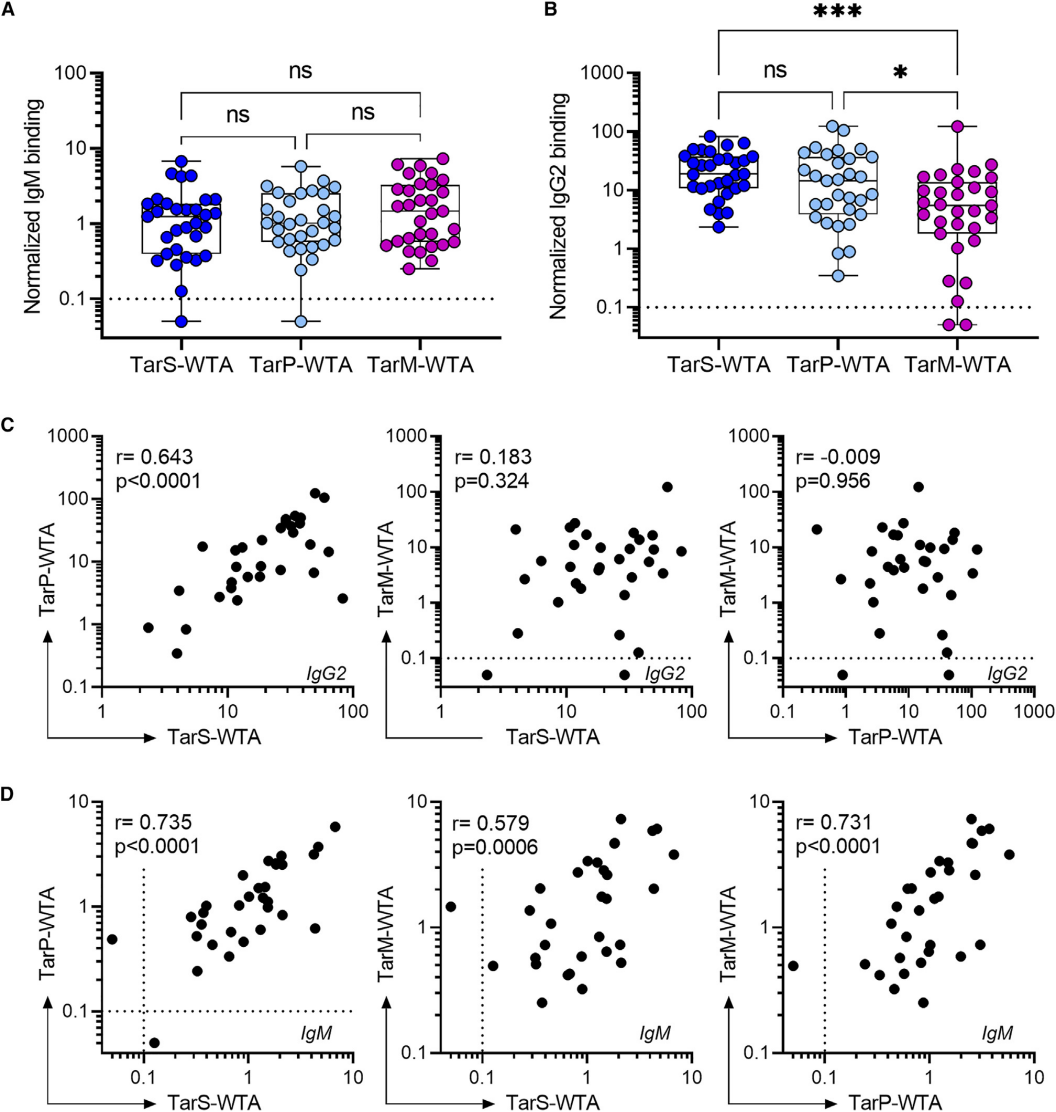
IgM and IgG2 antibody responses against three WTA glycotypes in healthy subjects (A and B) Normalized binding of (A) IgM and (B) IgG2 to beads coated with TarS-WTA, TarP-WTA, and TarM-WTA in plasma from healthy donors (*n* = 31). Boxplots extend from the 25th to 75th percentiles,the line represents the median, and the whiskers indicate the total range. Differences in antibody responses to the three WTA glycotypes were analyzed with Kruskal-Wallis test with Dunn’s correction. (C, D) Spearman correlations between binding of (C) IgG2 and (D) IgM reactivity toward distinct WTA glycotypes within individual donors. Each dot represents an individual donor (*n* = 31). Dotted line represents the lower limit of quantification, symbols shown below this line represent extrapolated values. ∗*p* < 0.05, ∗∗∗*p* < 0.001. See also Figure S2.

We employed a bead-based assay, adapted for multiplexing, using synthetic WTA oligomers to study the antibody repertoire (IgG1, IgG2, IgG3, IgM, and IgA) targeting specific *S. aureus* WTA glycotypes (Figure S1).^18^ Analysis of plasma samples from 31 healthy donors revealed specific IgM responses to all three WTA glycotypes in 29 out of 31 (94%) donors (Figure 1A). Furthermore, WTA-specific IgG2 antibodies were detected against at least two of the three WTA glycotype in all donors (Figure 1B) with 94% of individuals having detectable IgG2 levels against all three WTA glycotypes. Conversely, only low levels of WTA-specific IgA, IgG1, and IgG3 were observed in approximately half of the donors (Figure S2). IgG2 responses to TarS-modified WTA were consistently high and not significantly different from IgG2 levels against TarP-modified WTA (Figure 1B), likely due to cross-reactive antibody clones recognizing β-GlcNAc-WTA (Figure 1C).^18^ In contrast, IgM antibodies demonstrated a significant positive correlation in binding to all three WTA glycotypes, indicating the presence of poly-reactive IgM clones that bind GlcNAcylated WTA irrespective of GlcNAc configuration or linkage position (Figure 1D). These observations highlight the extensive and diverse antibody repertoire targeting *S. aureus* WTA in healthy individuals, with potential variations in binding specificities and characteristics across different antibody isotypes (IgG and IgM).

### WTA-specific IgM contributes to protection against *S. aureus* infection *in vitro* and *in vivo*

**Figure 2:**
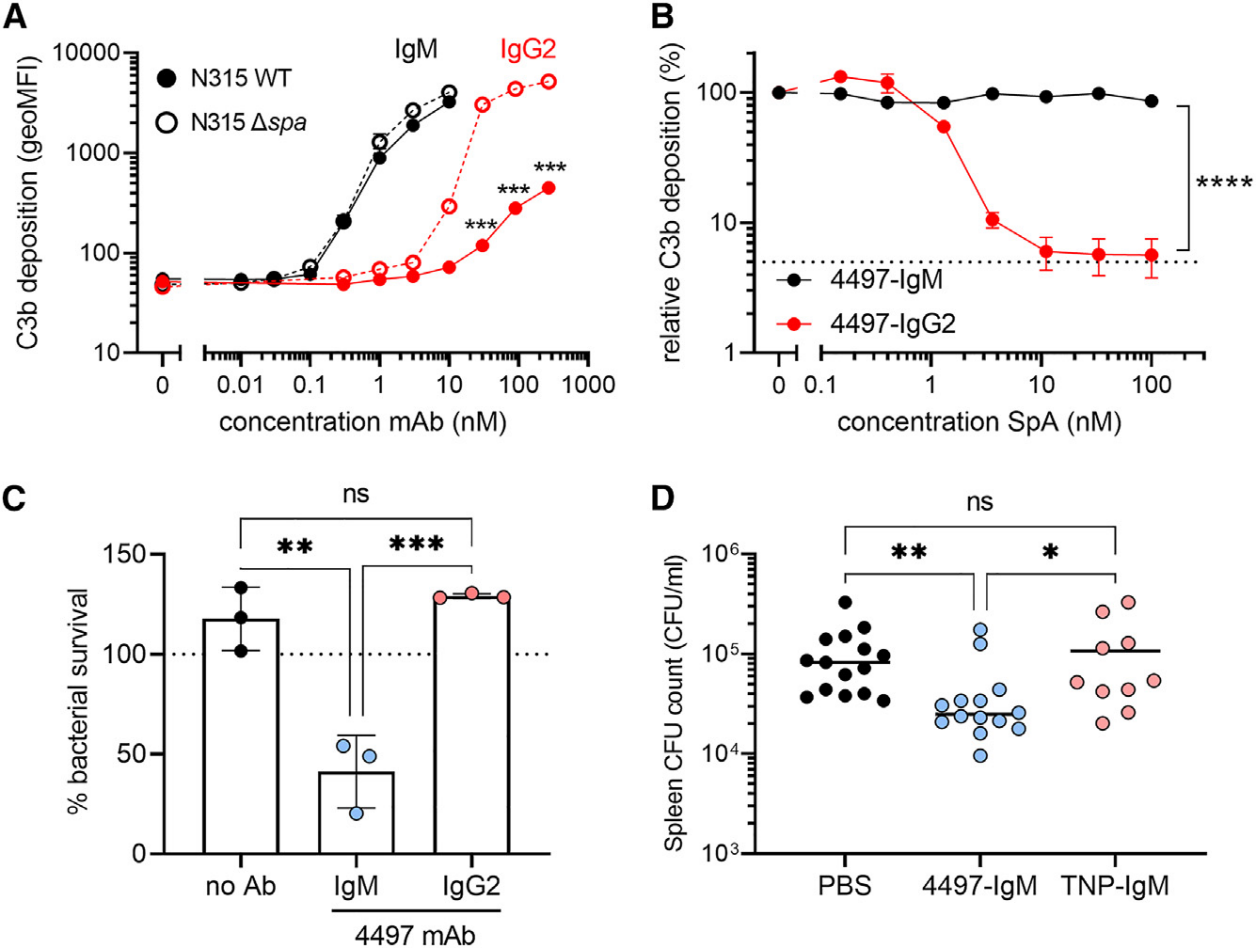
WTA-specific IgM supports immune-mediated clearance of *S. aureus in vitro* and in mouse infection experiments (A) Complement C3b deposition on *S. aureus* N315 wild-type (WT) or staphylococcal protein A-deficient (Δ*spa*) bacteria, pre-opsonized with 4497-IgG2 (0.3–270 nM) or 4497-IgM (0.01–10 nM). Error bars indicate SD of the mean of biological triplicates. Multiple unpaired t tests with Holm-Šídák correction were used to compare antibody-mediated C3b deposition on N315 WT and Δ*spa* for either IgM or IgG2. (B) Relative complement C3b deposition on SpA-deficient *S. aureus* (Newman Δ*spa/sbi*), pre-opsonized with 4497-IgG2 (10 nM) or 4497-IgM (1 nM) in presence of recombinant protein A (SpA, 0.15–100 nM). C3b deposition in the absence of SpA was set at 100%; the dotted line represents background C3b deposition without antibody opsonization. Error bars indicate SEM of the mean of biological triplicates. Differences between 4497-IgM and 4497-IgG2 were assessed using a two-way ANOVA with Šídák correction. (C) Neutrophil opsonophagocytic killing (OPK) of *S. aureus* N315 WT, in presence of 10 nM 4497-IgM, 10 nM 4497-IgG2, or no antibody control. Bacterial survival has been normalized over inoculum (dotted line at 100%); data are shown from three independent donors (mean + SD). Statistical analysis was performed using a one-way ANOVA with Tukey correction. (D) Spleen CFU counts from mice (*n* = 10–15 per group) at 24 h post infection with *S. aureus* N315 WT, passively immunized with 30 μg 4497-IgM, anti-TNP-IgM, or PBS control. Data were pooled from three independent experiments. Statistical analysis was performed using a Kruskal-Wallis test with Dunn’s correction. ns, non-significant, ∗*p* < 0.05, ∗∗*p* < 0.01, ∗∗∗*p* < 0.001, ∗∗∗∗*p* < 0.0001. See also Figure S3.

We postulated that WTA-specific IgM could play a more prominent role in protective immunity against *S. aureus* than IgG2, since the effector functions of opsonic IgG antibodies can be subverted by the well-characterized *S. aureus* virulence factor SpA. To this end, we compared the efficacy of IgM and IgG2 monoclonal antibodies (mAbs) derived from an original IgG1 clone targeting β-GlcNAc WTA (clone 4497).^17^^,^^28^ The IgM variant, 4497-IgM, was over 100-fold more effective compared to 4497-IgG2 at inducing complement C3b deposition at equimolar concentrations on *S. aureus* N315, which expresses β1,3- and β1,4-GlcNAc corresponding to the genetic presence of *tarP* and *tarS*, respectively (Figure 2A). Importantly, the presence of surface-bound (Figure 2A) or secreted SpA (Figure 2B) did not interfere with IgM-mediated C3b deposition, but did attenuate IgG2-mediated functions (Figures 2A and 2B). Furthermore, complement deposition by 4497-IgM triggered neutrophil-mediated killing of *S. aureus*, whereas 4497-IgG2 did not show this effect at equimolar concentrations (Figure 2C).

To exclude strain-specific effects and assess the potential hindrance by α1,4-GlcNAc WTA glycosylation on 4497-IgM functionality in *tarM*-positive strains, we assessed 4497-IgM-mediated complement activation on four additional *tarS*/*tarM*-positive *S. aureus* strains belonging to three different clonal complexes (Figures S3A and S3B). 4497-IgM was equally effective on α/β-GlcNAc-WTA expressing strains (Newman, MW2, NRS384, 8325-4) compared to the β-GlcNAc-WTA expressing strain N315, demonstrating that complement deposition is enhanced by WTA-specific IgM independent of WTA glycosylation phenotype or genetic lineage. Similar to SpA, the absence of IgG binding protein Sbi did not affect 4497-IgM-mediated complement deposition on *S. aureus* (Figures S3C and S3D).

To evaluate the protective potential of IgM *in vivo*, mice were passively immunized with 4497-IgM prior to *S. aureus* infection. The 4497-IgM-treated mice showed significantly lower colony-forming unit (CFU) counts in the spleen compared to control groups treated with PBS or a non-specific IgM antibody (Figure 2D). CFU counts were also lower in the kidneys of 4497-IgM-treated mice, although this did not reach statistical significance (Figure S3E). Possibly, this difference in organ-specific *S. aureus* burden is related to the distribution of the IgM mAb, since WTA-specific IgM (4497-IgM) mainly localized in the spleen but not in the kidneys, whereas the non-specific IgM control antibody (anti-TNP IgM) showed the opposite distribution (Figure S3F). Overall, these findings strongly support the concept that WTA-specific IgM plays a critical role in conferring protection against disseminating *S. aureus* bacteremia, likely by enhancing opsonophagocytic killing of the pathogen by neutrophils, the key effector cells in innate antibacterial host defense.

### Patients with *S. aureus* bacteremia have low levels of WTA-IgM

*In vivo* animal models have a poor track record for predicting translational success in human vaccine and therapeutic antibody development for *S. aureus*.^29^^,^^30^ To avoid overreliance on experimental models, we further investigated the potential role of WTA-specific IgM antibodies in protective immunity in a cohort of 36 ICU patients with *S. aureus* bacteremia (Table 1) by assessing the natural repertoire of WTA-specific IgM and IgG antibodies and their respective correlation to clinical outcomes. Plasma from ICU patients with *Streptococcus pyogenes* bacteremia (*n* = 13) were included as bacteremia patient control group (Table 1).

**Table 1:** Patient characteristics of ICU patients with *S. aureus* or *S. pyogenes* bacteremia

Characteristic	*S. aureus* (*n* = 36)	*S. pyogenes* (*n* = 13)	*p* value^a^
Male, *n* (%)	26 (72)	8 (62)	0.500
Age, years mean (SD)	61 (16)	49 (17)	0.072
Admission type, *n* (%)			
Medical	21 (58.3)	4 (30.8)	0.114
Planned surgical	6 (16.7)	0 (0)	0.174
Emergency surgical	9 (25)	9 (69.2)	0.007
Site of infection, *n* (%)			
Pulmonary	4 (11.1)	2 (15.4)	0.649
Cardiovascular	9 (25)	2 (15.4)	0.703
Musculoskeletal	7 (19.4)	0 (0)	0.167
Surgical site	4 (11.1)	0 (0)	0.562
Abdominal	2 (5.6)	0 (0)	>0.999
Soft tissue	6 (16.7)	7 (53.8)	0.023
Central nervous system	0 (0)	2 (15.4)	0.066
Unknown	4 (11.1)	0 (0)	0.562
Comorbidities prior to ICU admission, *n* (%)			
Diabetes mellitus	8 (22.2)	0 (0)	0.090
Non-metastatic solid tumor	5 (13.9)	2 (15.4)	>0.999
APACHE IV score, median (range)	84 (44–162)	67 (29–162)	0.349
White cell count, median (range)	12.5 (4.9–36.5)	18 (1.3–28)	0.026
Platelet count (SOFA coagulation score), *n* (%)			
≥150 (0)	24 (66.7)	1 (7.7)	<0.001
<150 (1)	8 (22.2)	5 (38.5)	0.288
<100 (2)	3 (8.3)	3 (23.1)	0.321
<50 (3)	1 (2.8)	4 (30.8)	0.014
Septic shock/sepsis, *n* (%)	14 (38.9)	4 (30.8)	0.307
Length of ICU stay in days, mean (SD)	18 (19)	7 (7)	0.033
ICU mortality, *n* (%)	6 (16.7)	2 (15.4)	>0.999

a To compare both patient cohorts, continuous variables were analyzed with a Mann-Whitney test and categorical variables were assessed with a Fisher’s exact test.

**Figure 3:**
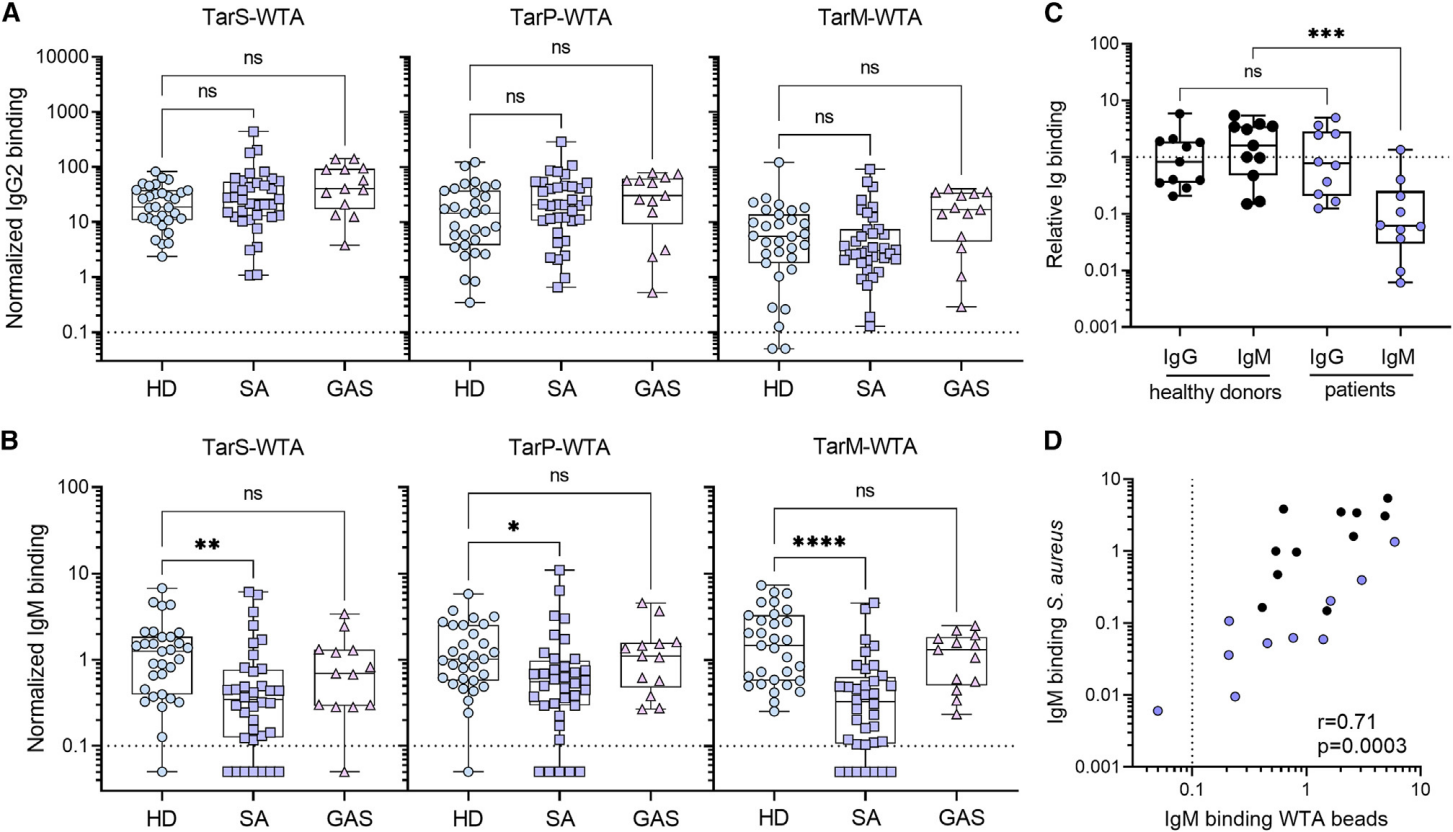
Patients with *S. aureus* bacteremia display reduced WTA-specific IgM antibody levels (A and B) Normalized binding of (A) IgG2 and (B) IgM to beads coated with TarS-WTA, TarP-WTA, and TarM-WTA. Boxplots represent data for healthy donors (HD, *n* = 31) and ICU patients with *S. aureus* (SA, *n* = 36) or *S. pyogenes* (GAS, *n* = 13) bacteremia and extend from the 25th to 75th percentiles. The line inside the box represents the median, whiskers indicate the total range with symbols representing individual donors. Statistical analysis was performed using a Kruskal-Wallis test with Dunn’s multiple comparison test to compare both patient cohorts with healthy donors. (C) IgG and IgM binding to *S. aureus* strain Newman Δ*spa/sbi* in sera from healthy donors (*n* = 11) and patients with *S. aureus* infection (*n* = 10), defined as relative binding compared to pooled human serum. Statistical analysis was performed using a Mann-Whitney test, to compare either IgG or IgM binding between healthy donors and patients. (D) Spearman correlation between IgM binding to Newman Δ*spa/sbi* and cumulative IgM binding to TarS- and TarM-WTA beads; patients (*n* = 10) are shown in blue and healthy donors (*n* = 11) in black. Dotted line represents the lower limit of quantification and symbols shown below the line represent extrapolated values. ns, non-significant, ∗*p* < 0.05, ∗∗*p* < 0.01, ∗∗∗∗*p* < 0.0001. See also Figure S4.

Our analysis focused on WTA-specific IgM and IgG2 responses using the same experimental setup as for the healthy donor group. We observed that IgG2 responses to any of the three WTA glycotypes were comparable between healthy donors and both patient groups (Figure 3A). In contrast, patients with *S. aureus* bacteremia exhibited significantly reduced IgM levels to all WTA glycotypes compared to healthy donors, with undetectable WTA-specific IgM in 7 out of 36 patients (19%) (Figure 3B). Notably, reduced WTA-specific IgM responses were not observed in patients with *S. pyogenes* bacteremia (Figure 3B), indicating a pathogen-specific phenomenon. While total IgM titers were generally lower in patients with *S. aureus* bacteremia compared to healthy controls, they were similar to patients with *S. pyogenes* bacteremia (Figures S4A and S4B) and did not correlate with WTA-specific IgM responses (Figure S4C). Moreover, IgM responses to CHIPS, a secreted and relatively conserved *S. aureus* virulence factor,^31^ were not significantly affected in the *S. aureus* bacteremia patient cohort compared to healthy donors and did not correlate with WTA-IgM reactivity or total IgM titers (Figures S4D–S4F). These findings suggest that the reduced WTA-specific IgM antibody levels in patients with *S. aureus* bacteremia cannot be attributed to a general decrease in overall IgM titers, and the effect appears to be antigen specific to some extent.

WTA is only one of many immunogenic antigens present on the *S. aureus* surface. To examine the impact of reduced WTA-IgM reactivity on IgM binding to the *S. aureus* surface, we used sera from a separate patient cohort with *S. aureus* bacteremia to determine IgM and IgG binding to *S. aureus* strain Newman (*tarS/tarM* positive). This strain was genetically deleted for *spa* and *sbi* to allow the assessment of both IgM and IgG binding without interference by IgG Fc-binding proteins. Our analysis revealed significantly lower IgM binding to intact *S. aureus* in sera from patients with *S. aureus* bacteremia compared to healthy donors, while no differences were observed for IgG binding (Figure 3C). Moreover, we observed a significant positive correlation between IgM binding to WTA beads and intact *S. aureus*, suggesting that WTA is a major target for IgM on the *S. aureus* surface (Figure 3D).

### WTA-specific IgM levels are inversely associated with disease mortality risk

**Figure 4:**
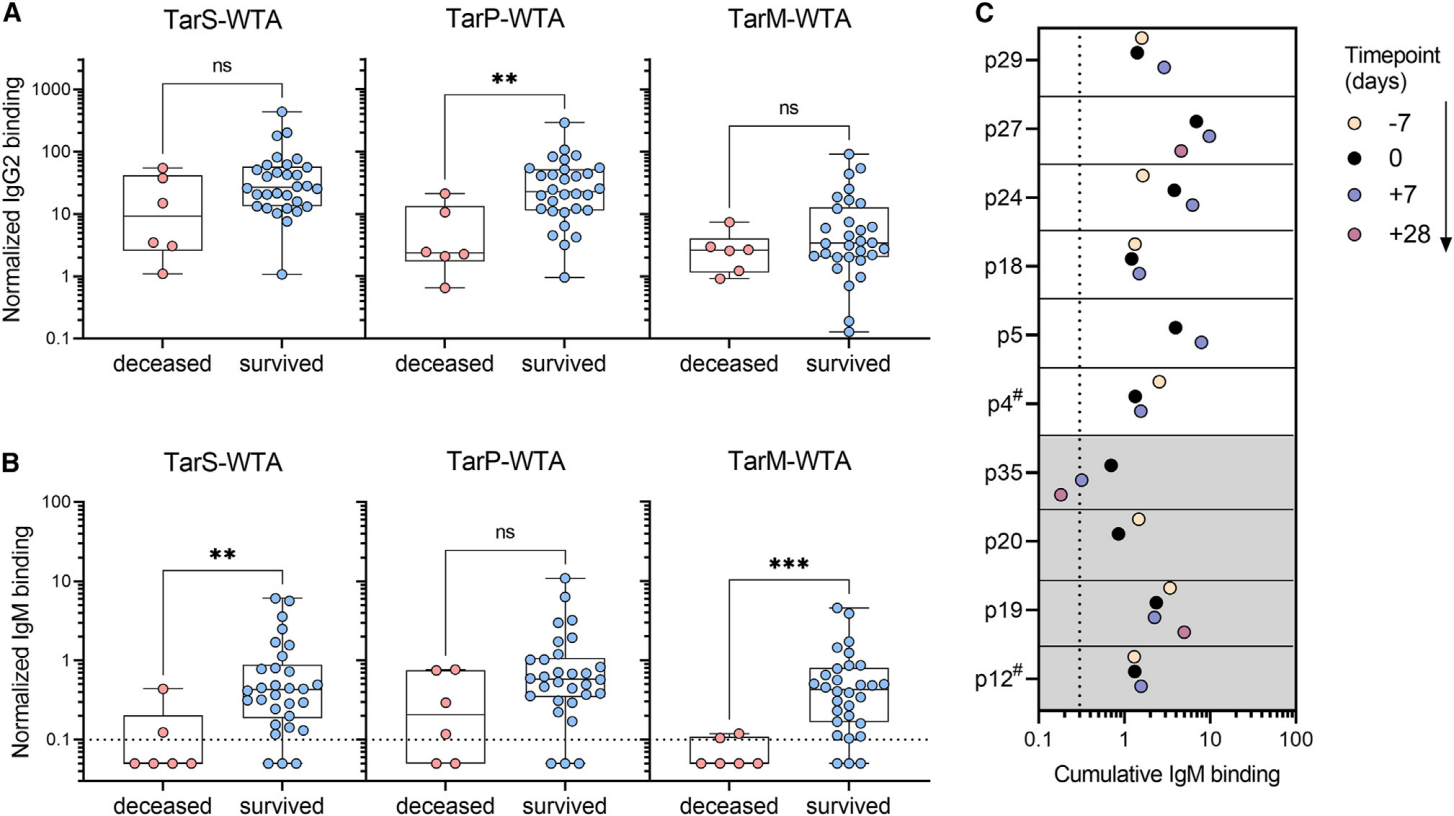
Association between longitudinal WTA-specific antibody responses and ICU mortality in patients with *S. aureus* bacteremia (A and B) Normalized binding of plasma-derived WTA-specific (A) IgG2 and (B) IgM according to the three WTA glycotypes from ICU patients with *S. aureus* bacteremia, stratified by ICU mortality. Boxplots extend from the 25th to 75th percentiles, and whiskers represent the total range. Symbols indicate individual patients with *S. aureus* infection (*n* = 36). Statistical analysis was performed using a Mann-Whitney test. (C) Cumulative IgM binding for the three WTA glycotypes (TarS-, TarP-, and TarM-WTA) in longitudinal plasma samples (in days from positive blood culture) for ten patients with *S. aureus* bacteremia. Plasma samples at time point zero coincides with plasma samples shown in Figures 3A/B and 4A/B. The shading indicates patients that died in the ICU (p12, p19, p20, and p35). # refers to a different timing of sample acquisition (not −7 days). Dotted line represents the lower limit of quantification and symbols shown below the line represent extrapolated values. See also Figure S5. ns, non-significant, ∗*p* < 0.05, ∗∗*p* < 0.01.

We next wondered whether low IgM reactivity to WTA within the patient cohort would correlate with a poor disease outcome, due to an impaired ability of the immune system to eradicate *S. aureus*. To examine a possible association between WTA-specific IgM levels and clinical outcomes, specifically mortality, WTA-specific antibody responses were stratified based on patient mortality. Among the 36 patients in the cohort, 6 (17%) died while in the ICU (Table 1). IgM levels, but not IgG2 levels, against TarS- and TarM-modified WTA were significantly lower in deceased patients (Figures 4A and 4B). For TarP-modified WTA, only IgG2 levels showed a significant reduction in deceased patients (Figure 4A), whereas IgM responses were not significantly different (Figure 4B). There were no significant differences in total IgM or IgG levels, age, length of ICU stay, or underlying co-morbidities between surviving or deceased patients (Figure S5A).

To determine whether the reduced WTA-IgM reactivity observed in patients with *S. aureus* bacteremia was a consequence of systemic infection or already existed prior to bacteremia, we analyzed longitudinal samples from 10 patients taken approximately one week before, one week after, and four weeks after the initial positive blood culture (dependent on sample availability). While a few patients showed minor fluctuations in WTA-specific IgM levels over the course of infection (e.g., patients 24 or 35), the responses to different WTA glycotypes remained relatively constant over time (Figures 4C and S5B). Notably, for two patients (indicated by #), the available earlier time point was not one week but three years (patient 4) and three months (patient 12) prior to the initial tested sample, showing consistent WTA-specific IgM responses over extended periods of time. Finally, three patients who later succumbed to *S. aureus* bacteremia already exhibited low WTA-specific IgM responses before the onset of bacteremia, indicating that low WTA-IgM reactivity may predispose to poor disease outcome.

### Low WTA-IgM reactivity impairs complement deposition on live *S. aureus* bacteria

**Figure 5:**
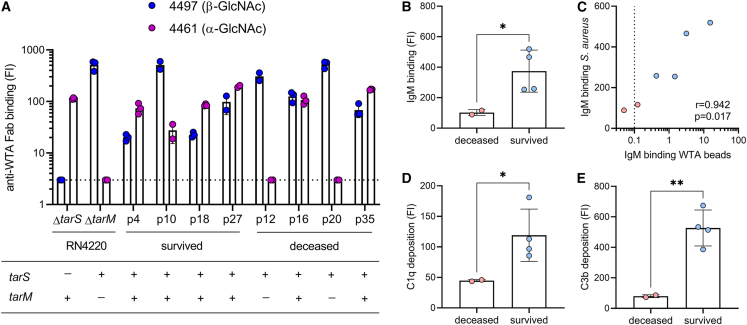
Complement deposition on *S. aureus* by WTA glycoprofile-specific IgM is reduced in ICU patients that succumbed to *S. aureus* bacteremia (A) Expression of β-GlcNAc-WTA (Fab clone 4497) and α-GlcNAc-WTA (Fab clone 4461) by clinical isolates from eight patients (survived patients: p4, p10, p18, p27; deceased patients: p12, p16, p20, p35) and reference strains RN4220 Δ*tarS* and Δ*tarM*. Below the corresponding WTA glycosyltransferase genotypes (*tarS*, *tarM*) as determined by PCR analysis. The dotted line indicates background staining. (B) IgM binding to *S. aureus* strain Newman Δ*spa/sbi* in 1% plasma from six patients infected with a *tarS*^+^*tarM*^+^*S. aureus* isolate (as shown in A), stratified on ICU mortality. (C) Spearman correlation between IgM binding to *S. aureus* (shown in A) and cumulative IgM binding to TarS-WTA and TarM-WTA beads in six patients, with deceased patients shown in red and survived patients in blue. Dotted line represents the lower limit of quantification and symbols shown below the line represent extrapolated values. (D and E) Deposition of (d) C1q and (e) C3b on Newman Δ*spa/sbi*, pre-opsonized with 3% patient plasma, stratified on ICU mortality. Anti-WTA Fab staining, deposition of IgM, C1q and C3b on *S. aureus* bacteria is depicted as geometric mean fluorescence intensity (FI) (mean + SD of biological duplicates or triplicates), each symbol indicates individual patients. Statistical analysis for C, D, and E was performed using unpaired t tests with Welch correction. ∗*p* < 0.05, ∗∗*p* < 0.01. See also Figure S6.

Our findings suggest that low WTA-specific IgM responses are associated with increased disease mortality in patients with *S. aureus* bacteremia. Since IgM-mediated complement activation is not affected by SpA and plays a critical role in *S. aureus* killing (Figure 2), we hypothesized that impaired bacterial opsonization due to low WTA-specific IgM responses could contribute to this association within the patient cohort. Before addressing this experimentally, we first determined WTA glycosylation genetically and phenotypically in available clinical isolates to align the patient’s antibody response with the WTA glycoprofile of the infecting *S. aureus* strain. Confirmation of the expressed glycoprofile with GlcNAc-WTA-specific Fab fragments is key, since we recently showed that some *S. aureus* strains harbor inactivating mutations of the encoded Tar enzymes.^23^ We obtained original isolates from eight patients within the *S. aureus* ICU cohort; four that survived the infection (patient 4, 10, 18, and 27) and four that passed away in the ICU (patient 12, 16, 20, and 35). All eight *S. aureus* isolates contained *tarS* and expressed the corresponding β1,4-GlcNAc moiety on their surface (Figure 5A); six isolates also contained *tarM* and showed corresponding surface expression of α1,4-GlcNAc-WTA (Figure 5A). Of the six patients that had been infected by *tarS*/*tarM*-expressing *S. aureus* strains, four survived the infection (patient 4, 10, 18, and 27) and two passed away (patient 16 and 35) (Figure 5A). IgM binding and C1q- and C3b-deposition on the surface of a *tarS*/*tarM*-positive *S. aureus* strain (Newman Δ*spa/sbi*) were significantly reduced when incubated with plasma from deceased patients compared to surviving patients (Figures 5B–5E). IgM binding to *S. aureus* strongly correlated with IgM binding to WTA beads (Figure 5C). Furthermore, spiking patient plasma samples with low WTA-specific IgM with 4497 IgM mAb boosted C3 deposition on *S. aureus* in 4 out of 7 samples (Figure S6). In contrast, we did not observe this effect in patient plasma samples with high WTA-specific IgM (Figure S6). These results reinforce the importance of glycan-specific IgM binding and subsequent complement C3b deposition on the *S. aureus* surface for patient survival following bloodstream infection.

## Discussion

In this study, we conducted antibody profiling for glycosylated *S. aureus* RboP-WTA, a prominent glycopolymer expressed by nearly all *S. aureus* strains.^23^^,^^32^ For this work, we took advantage of synthetic glycosylated RboP-WTA oligomers, which resemble *S. aureus*-expressed WTA as previously confirmed by WTA-specific mAbs and recognition by the C-type lectin receptor langerin.^18^^,^^33^ Our data corroborated the near-universal presence of WTA-specific IgM and IgG antibodies in healthy individuals,^17^^,^^25^ confirming widespread exposure to the bacterium in the absence of clinical disease.^18^ Remarkably, IgM outperformed IgG in complement activation and conferred protection against systemic *S. aureus* infection in mice, while being unaffected by the major *S. aureus* virulence factor SpA. Importantly, WTA-specific IgM levels, but not IgG or IgA, were significantly lower among patients with *S. aureus* bacteremia, particularly in non-survivors, and correlated with reduced complement activation, a vital response for recruiting and activating neutrophils.^6^^,^^34^ Our findings suggest that IgM plays a critical role in protecting against severe *S. aureus* infections, challenging the notion that it is merely a transitional antibody isotype.^16^ Further experimental and clinical studies are needed to determine the generalizability of our findings. Experimental studies may include the protective capacity of WTA-specific IgM in different mouse infection models, e.g., infecting with different *S. aureus* strains via various infection routes and assessing multiple parameters for infection severity. From a clinical perspective, the inclusion of other patient cohorts, for example children or patients with *S. aureus* pneumonia or severe skin and soft tissue infection, may be employed to investigate whether insufficient opsonic IgM antibodies targeting WTA or other surface antigens represent a risk factor for poor clinical outcomes.

Our findings may have important implications for clinical care management, particularly in risk assessment and prophylaxis for hospitalized patients. Previous case reports have indicated that patients with primary selective IgM deficiency are more susceptible to *S. aureus* infections,^35^^,^^36^ and reduced IgM responses can also occur in individuals with advancing age or impaired splenic function.^37^^,^^38^ Based on our data, low WTA-specific IgM titers may be a risk factor for the development of *S. aureus* bacteremia and poor clinical outcomes. Conceivably, patients with a planned procedure that holds risk for opportunistic *S. aureus* infections could be evaluated for WTA-specific IgM titers, similar to the assessment of other risk factors such as neutrophil counts and *S. aureus* colonization,^39^^,^^40^ which clinicians could weigh in decisions on antibiotic selection or frequency of monitoring. Moreover, future studies could explore the potential prophylactic or therapeutic application of polyclonal (e.g., IgM-enriched intravenous immunoglobulin (IVIg)^41^^,^^42^) or monoclonal IgM to improve outcomes in *S. aureus* bacteremia. Here, we provide a first proof of concept that the administration of opsonic IgM mAbs to patients with low endogenous levels of WTA-specific IgM may improve anti-*S. aureus* immunity, although further investigation is warranted to determine the breadth of protection. Indeed, a limited number of promising preclinical proof-of-principle studies have been reported for pathogen-targeted therapeutic monoclonal IgM antibodies against influenza,^43^ severe acute respiratory syndrome coronavirus 2,^44^ and *Neisseria meningitidis*.^45^

The potential advantages of opsonic IgM over IgG in protecting against *S. aureus* bacteremia may have implications for vaccine development. Current vaccination strategies for *S. aureus* have focused on inducing high levels of IgG, the classic opsonic antibody isotype, but these approaches have consistently failed in human clinical trials, despite promising results in preclinical animal studies. The effectiveness of IgG-mediated strategies may be hindered by the presence of IgG-Fc binding proteins such as SpA and Sbi, which effectively interfere with IgG-mediated downstream functions. Our data from a cohort of patients with *S. aureus* bacteremia suggest that efforts to boost durable opsonic IgM responses through vaccination may be more successful, partly because SpA does not interfere with IgM-mediated effector functions like it does with IgG. Characterizing the B cells responsible for producing these glycan-specific IgM antibodies, as well as understanding how to induce their production, will advance our fundamental understanding of protective adaptive immunity in humans. These insights could likewise inform vaccine development efforts for other human bacterial pathogens that express IgG-Fc or IgA-Fc binding immune evasion factors on their surfaces, including *S. pyogenes*.^46^

In conclusion, we provide evidence of a key role for opsonic IgM in protective immunity to *S. aureus*. The manipulation of B cell responses by pathogens to their own advantage is an area of increasing research interest,^47^ including the observation that Fc binding by SpA *in vivo* can trigger large-scale supraclonal B cell depletion by VH-targeted activation-induced cell death in mouse models.^48^^,^^49^ Further research to dissect the host-pathogen interactions dictating opsonic IgM responses to *S. aureus* can contribute to risk stratification of hospitalized patients and/or rational design of antibody-based therapies and vaccines against this foremost human pathogen.

### Limitations of the study

There are limitations to our study, which warrant caution and require future investigation. Firstly, the experimental mouse infection model does not resemble infections in humans, in terms of host immune status, route of infection, and bacterial inoculum. Secondly, the IgM mAb (clone 4497) used in this study is derived from an IgG1 clone, and therefore likely is not representative of circulating human WTA-specific IgM antibodies. The third limitation is the small group size of healthy donors and patients. Future studies should include larger groups of patients varying in *S. aureus* infection severity, using data from this pilot study for sample size calculations. Fourthly, we were not able to expand on functional assays using patient material, e.g., assessing opsonophagocytic killing of *S. aureus* in patients with low WTA-specific IgM levels, due to limited sample availability. Finally, a prospective study would be required to validate the hypothesis that low WTA-specific IgM levels increase the risk of *S. aureus* bacteremia and mortality.

## Resource availability

### Lead contact

Further information and requests for resources and reagents should be directed to the lead contact Nina M. van Sorge (n.m.vansorge@amsterdamumc.nl).

### Materials availability

This study did not generate new unique reagents.

### Data and code availability

All data reported in this paper will be shared by the lead contact upon request, depending on patient confidentiality and deductive disclosure issues. This paper does not report original code. Any additional information required to reanalyze the data reported in this paper is available from the lead contact upon request.

## Acknowledgments

We kindly thank Thilo Stehle for providing recombinant TarP enzyme, Andreas Peschel for the bacterial strains, Alex Stream and Elisabet Bjanes for human neutrophil isolation, Chih-Ming Tsai for support with the *in vivo* experiments, and András Spaan and Pieter-Jan Haas for assistance in obtaining the clinical isolates. We also thank Kok van Kessel, Bart Bardoel, Yvonne Pannekoek, Robin Temming, and Rob van Dalen for the valuable discussions. This work was supported by the Vidi (91713303) and Vici (09150181910001) research program to N.M.v.S., which is financed by the Dutch Health Council (NWO), and the BactiVac Training grant for sponsoring A.H. to visit UCSD. The work of P.F.K. was funded by Genmab B.V.

## Author contributions

A.H. and N.M.v.S. conceptualized and designed the project, with valuable input from J.A.G.v.S., S.H.M.R., V.N., and G.Y.L.; key resources were provided by S.A., J.D.C.C., C.J.C.d.H., P.C.A., M.R.J.V., J.L.G.H.M., T.t.D., T.W.v.d.V., O.L.C., M.J.M.B., V.N., and G.Y.L.; A.H. and P.F.K. conducted the experiments; and A.H. and N.M.v.S. wrote the manuscript with input from all authors.

## Declaration of interests

The authors declare no competing interests.

## STAR★Methods

### Key resources table

**Table:** 

REAGENT or RESOURCE	SOURCE	IDENTIFIER
**Antibodies**		

Alexa Fluor 647-conjugated goat F(ab’)2 -anti human kappa	Southern Biotech	Cat # 2062-31; RRID: AB_2795742
PE-conjugated goat anti-human IgG	Southern Biotech	Cat # 2040-09; RRID: AB_2795648
FITC-conjugated goat anti-human IgM	Southern Biotech	Cat # 2020-02; RRID: AB_2795600
Alexa Fluor 647-conjugated goat F(ab’)2 anti-human IgA	Southern Biotech	Cat # 2052-31; RRID: AB_2795711
PE-conjugated mouse anti-human IgG1 Fc	Southern Biotech	Cat # 9054-09; RRID: AB_2796628
Alexa Fluor 488-conjugated mouse anti-human IgG2 Fc	Southern Biotech	Cat # 9070-30; RRID: AB_2796640
Alexa Fluor 647-conjugated mouse anti-human IgG3 hinge	Southern Biotech	Cat # 9210-31; RRID: AB_2796703
FITC-conjugated rabbit F(ab’)2 anti-human C3c	Dako, Cruz et al.^13^	Cat #F0201; RRID: AB_2335709
FITC-conjugated rabbit F(ab’)2 anti-human C1q	Dako, Cruz et al.^13^	Cat #F025402
PE-conjugated goat F(ab’)2 anti-human IgM	Southern Biotech	Cat # 2022-09; RRID: AB_2795614
Alexa Fluor 647-conjugated goat anti-human IgG	Southern Biotech	Cat # 2040-31; RRID: AB_2795651
sheep anti-human IgG	ICN Biomedicals	Cat # 682311; RRID: AB_2335117
sheep anti-human IgM	ICN Biomedicals	Cat # 682331
ChromPure human IgG	Jackson Immunoresearch	Cat # 009-000-003; RRID: AB_2337043
ChromPure human IgM	Jackson Immunoresearch	Cat # 009-000-012; RRID: AB_2337048
horseradish peroxidase (HRP)-conjugated goat anti-human IgG	Southern Biotech	Cat # 2040-05; RRID: AB_2795644
horseradish peroxidase (HRP)-conjugated goat anti-human IgM	Southern Biotech	Cat # 2020-05; RRID: AB_2795603
Bacterial strains		
*Staphylococcus aureus* N315 wildtype (WT)	NARSA strain collection	www.narsa.net
*S. aureus* N315 Δ*spa*	Gerlach et al.^22^	N/A
*S. aureus* Newman WT	ATCC	Cat # 13420
*S. aureus* Newman Δ*spa*Δ*sbi*	Sibbald et al.^50^	N/A
*S. aureus* Newman Δ*spa*Δ*sbi* - mAmetrine	Cruz et al.^12^	N/A
*S. aureus* MW2 WT	CDC^51^	N/A
*S. aureus* NRS384 WT	NARSA strain collection	www.narsa.net
*S. aureus* NRS384 Δ*tarM*	Winstel et al.^32^	N/A
*S. aureus* 8325-4 WT	NARSA strain collection	www.narsa.net
*S. aureus* RN4220 Δ*tarS*	Winstel et al.^52^	N/A
*S. aureus* RN4220 Δ*tarM*	Winstel et al.^52^	N/A
*S. aureus clinical isolates*	This study	N/A

**Biological samples**		

IgG- and IgM-depleted human serum	Zwarthoff et al.^53^	N/A
Baby rabbit serum	Pel Freez	Cat # 31061
Bovine serum albumin	Serva	Cat # 11930
Human serum albumin (Albuman)	Prothya Biosolutions	RVG: 103585

**Chemicals, peptides, and recombinant proteins**		

UDP-GlcNAc	Merck	Cat #U4375
protein A from *S. aureus*	Cruz et al.^13^	N/A
CHIPS from *S. aureus*	De Haas er al.^31^	N/A
TMB substrate for ELISA	Sigma	Cat #T0440
RPMI Medium 1640(1x)	ThermoFisher Scientific	Cat # 52400-025
Polymorphprep	Alere Technologies	Cat # 1114683
Achromopeptidase	Sigma	Cat # A3547
Lysostaphin	Sigma	Cat #L9043
PEI max	Polysciences	Cat # 24765
POROS™ CaptureSelect™ IgM Affinity Matrix	ThermoFisher Scientific	Cat # 2812892005
Jacalin Agarose	ThermoFisher Scientific	Cat # 20395

**Critical commercial assays**		

NucleoBond Xtra Midi kit	Macherey-Nagel	Cat # 740410

**Experimental models: Organisms/strains**		

*Mus Musculus* BALB/c	Charles River	Strain # BALB/cAnNCrl

**Oligonucleotides**		

Forward TarS: 5′- GTGAACATATGAGTAGTGCGTA-3′	Integrated DNA Technologies	N/A
Reverse TarS: 5′-CATAATGTCCTTCGCCAATCAT-3′	Integrated DNA Technologies	N/A
Forward TarM: 5′-GGGATACCCATATATTTCAAGG-3′	Integrated DNA Technologies	N/A
Reverse TarM: 5′- CAATTCGCTTCGTTGGTACCATTC-3′	Integrated DNA Technologies	N/A

**Software and algorithms**		

GraphPad Prism 10	GraphPad Software	https://www.graphpad.com/scientificsoftware/prism/
FlowJo (v.10.8.1)	FlowJo	https://www.flowjo.com/

**Other**		

Dynabeads™ M-280 Streptavidin	ThermoFisher Scientific	Cat # 11205D

### Experimental model and study participant details

#### Human participants

Blood from healthy individuals (*n* = 31) was collected in EDTA tubes with full informed consent and approval from the Institutional Review Board of the University Medical Center Utrecht (METC protocol 07–125/C, approved March 1, 2010) in accordance with the Declaration of Helsinki. EDTA-plasma was obtained by centrifugation (10 min, 2,000 *g* at 4°C), and stored at −80°C.

Patients, admitted to the intensive care unit (ICU) were included based on specific inclusion criteria as part of the Molecular Diagnosis and Risk Stratification of Sepsis (MARS) study (ClinicalTrials.gov, NCT01905033). The Institutional Review Board approved an opt-out consent method (protocol number 10-056C). Plasma samples were collected from leftover blood drawn for routine care in EDTA-treated tubes on day one after a positive *S. aureus* (*n* = 36) or *Streptococcus pyogenes* (*n* = 13; all patients available in cohort) blood culture and stored at −80°C within 4 h after collection. Information on patient characteristics, including age, gender and clinical parameters, is indicated in Table 1.

Blood from patients with *S. aureus* bacteremia (*n* = 10, METC protocol 19/495) and healthy donors (*n* = 11, METC protocol 07–125/C) were collected in serum tubes and centrifuged for 10 min at 3,000 rpm, 4°C. Serum samples were aliquoted and stored at −80°C. Samples were received within 1 h of blood draw and processed immediately. Human serum samples were only used for data shown in Figures 3C and 3D, all other figures depict data from human EDTA-plasma samples.

For neutrophil killing experiments, human blood was collected after informed consent from healthy human volunteers as approved by the University of California San Diego (UCSD) Human Research Protection Program.

For all human samples excluding the patients included in the MARS cohort, there is no personal information (e.g., age, gender) available, limiting the generalizability of the study.

#### Mouse strains

Mouse studies were reviewed and approved by the Institutional Animal Care and Use Committee. Experiments were performed in accordance with regulations of the Animal Care Program at University of California, San Diego. Six weeks old BALB/c mice were purchased from Charles River Laboratories. All mice were female, and were housed in specific-pathogen free facilities at University of California, San Diego. Age-matched mice were used for *in vivo* experiments.

#### Bacterial culture conditions

Bacterial strains, used in this study, are listed in the key resources table. Bacteria were cultured overnight in 3 mL Todd-Hewitt broth (THB; Oxoid) at 37°C with agitation. For Newman Δ*spa/sbi* mAmetrine,^12^ bacteria were grown in the presence of 10 μg/mL chloramphenicol. The following day, overnight cultures were subcultured in fresh THB and grown until reaching mid-exponential growth phase, corresponding to an optical density of 0.5–0.6 at 600 nm (OD600). Bacteria were collected by centrifugation (10 min @ 3,000g, 4°C) and resuspended in PBS 0.1% BSA to an OD_600_ of 0.4 for WTA glycoprofiling. For complement deposition and antibody binding experiments, bacteria were resuspended in RPMI supplemented with 0.05% human serum albumin (HSA) or 0.1% BSA (RPMI-A) to an OD600 of 0.4–0.5, and stored at −20°C until further use.

### Method details

#### Glycosylation and coating of WTA beads

Biotinylated RboP-WTA hexamers were synthesized and glycosylated using recombinant TarS, TarP, and TarM enzymes as previously described.^18^^,^^22^^,^^33^^,^^54^ Biotinylated RboP oligomers (0.17 mM) were incubated for 2 h at room temperature with the respective recombinant glycosyltransferase enzymes (6.3 μg/mL) and UDP-GlcNAc (2 mM, Merck) in glycosylation buffer (15 mM HEPES, 20 mM NaCl, 1 mM EGTA, 0.02% Tween 20, 10 mM MgCl2, 0.1% BSA, pH 7.4) (Figure S1A). The glycosylated RboP hexamers were then coupled to magnetic streptavidin (5× 10^7^) beads (Dynabeads M280 Streptavidin, Thermo Fisher Scientific) for 15 min at room temperature. The coated beads were washed three times with PBS 0.1% BSA 0.05% Tween 20 (PBS-BT) and stored at 4°C. The successful coating of WTA beads was validated by binding of monoclonal IgG1 antibodies (3 μg/mL) specific for α-GlcNAc-WTA (TarM-modified WTA, clone 4461), β-GlcNAc WTA (TarS- and TarP-modified WTA, clone 4497) and β-1,4-GlcNAc WTA (TarS-modified WTA, clone 6292), followed by detection with goat-anti human kappa-Alexa Fluor 647 (5 μg/mL, Southern Biotech) and analysis of geometric mean fluorescence intensity (geoMFI) by flow cytometry (BD FACSVerse).

#### Production of monoclonal antibodies

Monoclonal antibodies (mAbs) targeting β-GlcNAc-WTA (clone 4497) in different Ig isotypes and IgG subclasses were expressed in EXPI293F cells (Thermo Fisher) as previously described.^33^^,^^55^ Heavy chain (hG) and kappa light chain (hK) constant regions for human IgG1, IgG2, IgG3, IgM and IgA1 were cloned into the XbaI-AgeI cloning sites of the pcDNA34 vector (ThermoFisher), along with previously described variable regions (VH and VL) derived from patent WO 2014/193722 A1.50^17^^,^^28^ (Table S1). The VH and VL sequences, preceded by a Kozak sequence (ACCACC) and the HAVT20 signal peptide (MACPGFLWALVIST- CLEFSMA), were codon-optimized for human expression and synthesized as gBlocks (IDT). Gibson assembly was used to clone VH and VL gBlocks into the pcDNA34 vector, upstream of the Ig heavy chain (hG) and kappa light chain (hK) constant regions, following the manufacturer’s instructions. NheI and BsiWI were used as the 3′ cloning sites for VH and VL, respectively, to preserve the amino acid sequence of the immunoglobulin heavy and kappa light chains nce. For IgM, BamHI was used as the 3′ cloning site for VH. The constructs were transformed into E. coli TOP10F′ cells through heat shock, and clones were verified by PCR and Sanger sequencing (Macrogen). Plasmids were isolated using the NucleoBond Xtra Midi kit (Macherey-Nagel) and sterilized using 0.22 μm Spin-X centrifuge columns (Corning). For protein production, we used EXPI293F cells and their expression medium (Thermo Fisher); cells were cultured at 37°C, 8% CO2 in conical flasks with culture filter caps (Sigma) placed on a rotation platform (125 rotations/min). One day prior to transfection, the cells were diluted to a concentration of 2 x 10^6^ cells/mL, and 100 mL of cell culture was used for transfection the next day. In 10 mL of Opti-MEM (Thermo Fisher), 500 μL PEI-max (1 μg/μL; Polysciences) was mixed with DNA (1 μg/mL cells) in a 3:2 ratio of hK and hG vectors. After a 20 min incubation at room temperature, this DNA/PEI mixture was added dropwise to 100 mL of EXPI293F cells (2 × 10^6^ cells/mL). After 5 days, Ig expression was verified by SDS-PAGE, and the cell supernatant was collected by centrifugation and filtration through a 0.45 μM filter. IgG1 and IgG2 were purified using a HiTrap Protein A column (GE Healthcare) and Äkta Pure (GE Healthcare). Elution was performed in 0.1M citric acid, pH 3.0, and neutralization done with 1M Tris, pH 9.0. IgG3 was purified using a HiTrap Protein G column (GE Healthcare), with elution in 0.1M Glycine-HCl, pH 2.7, followed by neutralization with 1M Tris, pH 8.0. IgM purification involved dialysis against PBS, and additional NaCl was added to the IgM preparation to achieve a final concentration of 500 mM before application to a POROS CaptureSelect IgM Affinity matrix (Thermo Scientific) column. IgM was eluted using 0.1M Glycine-HCL pH 3.0 on the ÄKTA Pure system. 0.5M NaCl was added to the pooled fraction, which was then neutralized with 1M Tris pH 7.5. For IgA1 purification, a Jacalin agarose (Thermo scientific) column was used, followed by elution with 0.1M Melibiose (Sigma). All Ig fractions underwent overnight dialysis in PBS at 4°C, and purified mAbs were stored at −20°C.

#### Antibody binding to WTA beads

To analyze the presence of WTA-specific antibodies in human plasma or sera, we incubated 1 x 10^5^ beads coated with glycosylated RboP-WTA oligomers (WTA beads) and non-coated beads with a 3-fold serial dilution range of human plasma or sera (concentrations ranging between 0.01% and 3%) in a 96-well round bottom plate (Greiner) at 4°C in PBS-BT for 20 min. The beads were washed once with PBS-BT using a plate magnet, incubated with a mixture of either (1) goat anti-IgG-PE, goat anti-IgM-FITC and goat F(ab)2 anti-IgA-Alexa Fluor 647 or (2) mouse anti-IgG1 Fc-PE, anti-IgG2 Fc-Alexa Fluor 488 and mouse anti-IgG3 hinge-Alexa Fluor 647 (1 μg/mL, all from Southern biotech) for an additional 20 min at 4°C. After another wash, the beads were analyzed by flow cytometry (BD FACSVerse) (Figure S1B) using a standardized template. We measured the geometric mean fluorescence intensity (geoMFI) of 10,000 events within a set gate (based on FSC-A/SSC-A).

Antibody binding to WTA beads was measured in triplicate or duplicate. The geoMFI values were corrected for median background binding to non-coated beads, except for analysis of sera from healthy donors and patients for technical considerations (Figures 3C and 3D). Background-corrected geoMFI values were interpolated using a standard curve of β-GlcNAc WTA specific mAb (clone 4497 in IgG1/IgG2/IgG3/IgM/IgA isotype, 0.003–10 μg/mL) binding to TarS-WTA beads (Figure S1C). For longitudinal sample analysis, 2xβ1,4-GlcNAc WTA coated beads^54^ were used for the 4497-mAb standard curve. Interpolated values were adjusted for the dilution factor, and the mean normalized antibody binding was calculated using values from at least two dilutions. Values equal to or lower than background were assigned a value of 0.05. Pooled human EDTA-plasma from nine healthy donors was included in each measurement as control sample, ensuring an inter-assay coefficient of variation (CV) < 25% (Figure S1D).

#### Complement deposition assay

To assess antibody-mediated complement deposition on intact *S. aureus*, bacteria (∼1 × 10^6^ CFU) were incubated with diluted monoclonal antibodies (IgM/IgG2) or human plasma (1:33 and 1:100) in RPMI-A for 30 min at 4°C. In case of data shown in Figure 2B, a titration of wild-type recombinant protein A (SpA-WT, 0.15–100 nM), produced as described in,^13^ was added simultaneously with 4497-IgM (1 nM) or 4497-IgG2 (10 nM). The bacteria were washed, collected by centrifugation, and incubated with 1% IgG- and IgM-depleted human serum^53^ for 30 min at 37°C in RPMI-A. Subsequently, the bacteria were washed with RPMI-A and incubated with rabbit F(ab’)_2_ anti-human C3c-FITC (also reactive with C3b), rabbit F(ab’)_2_ anti-human C1q-FITC (both at 5 μg/mL, Dako as described in^13^), or goat F(ab’)_2_ anti-human IgM-PE (5 μg/mL, Southern Biotech) for 30 min at 4°C. The bacteria were washed, fixed in 1% paraformaldehyde in RPMI, and analyzed by flow cytometry (BD FACSVerse or Canto) using a standardized template. We measured the geometric mean fluorescence intensity (geoMFI) of 10,000 events within a set gate (based on FSC-A/SSC-A).

#### Neutrophil opsonophagocytic killing assay

Human neutrophils were freshly isolated from whole blood using Polymorphprep (Alere technologies) per manufacturer instructions. Bacteria were opsonized with 10 nM 4497-IgM or 10 nM 4497-IgG2 monoclonal antibodies in the presence of 2% baby rabbit serum (Pel Freeze) in RPMI-A for 30 min at 37°C while shaking (650 rpm). Subsequently, freshly isolated human neutrophils were added at a ∼1:10 bacteria to cell ratio in triplicate and incubated for 60 min at 37°C with agitation (200 rpm). To release the internalized bacteria, neutrophils were lysed by incubating 15 min on ice with 0.3% (w/v) saponin (Sigma- Aldrich) in sterile water. Samples were serially diluted in PBS and plated on THA plates in duplicate. CFUs were counted after overnight incubation at 37°C, and percentage survival was calculated and normalized over inoculum.

#### Murine model of systemic *S. aureus* infection

Animal experiments were performed with *S. aureus* N315 to align with the bulk of *in vitro* experiments. *S. aureus* N315 WT was grown overnight from a freshly streaked blood agar plate, diluted 1:200 in THB and grown to an OD600 of 0.7, followed by two washes with PBS. Seven to nine-week old BALB/c female mice were anesthetized with isofluorane and injected intravenously with monoclonal IgM antibodies (30 μg in 150 μL PBS) by retro-orbital injection. After 3 h, mice were infected with *S. aureus* N315 (3 × 10^7^ CFU) by intra-peritoneal (i.p.) injection. Spleen and kidneys were harvested after 24 h post-infection, homogenized in phosphate-buffered saline (PBS), serially diluted and plated on THB agar plates for CFU enumeration.

We opted for this experimental design, where IgM was administrated intravenously and *S. aureus* intraperitoneally, to model the majority of clinical cases of *S. aureus* bacteremia (i.e., disseminated bacteremia) and to adhere to the imposed constraints of a single intravenous injection in a 24-h time window. Moreover, direct intravenous compared to intraperitoneal injection of IgM ensured an more accurate estimation of the amount of human IgM in the circulation,since no loss would occur due to barrier crossing. We choose a 3 h time interval between IgM mAb administration and *S. aureus* infection to accommodate experimental logistics and the relatively short t_1/2_ of human IgM in mice of ∼8 h.^56^

#### IgG and IgM binding to *S. aureus*

Newman Δ*spa*/*sbi* mAmetrine bacteria were diluted 50 times from frozen stocks (OD600 of 0.5), and incubated with a serial dilution of heat inactivated (56°C 30 min) human sera in RPMI-A for 30 min at 4 °C at 600 rpm to allow antibody binding. Heat-inactivated pooled human serum was taken as reference, setting binding of this sample to 1. The bacteria were washed, collected by centrifugation (7 min at 3,500 rpm, 4°C) and incubated with goat anti-human IgG-Alexa Fluor 647 and goat anti-human IgM-PE (both Southern Biotech) for 30 min at 4 °C at 600 rpm. The bacteria were washed and fixed in PBS 1% formaldehyde before analysis by flow cytometry (BD FACSVerse) using a standardized template. We measured the geometric mean fluorescence intensity (geoMFI) of 10,000 events within a set gate (based on FSC-A/SSC-A and mAmetrine).

#### WTA glycoprofiling of *S. aureus* isolates

DNA was isolated from *S. aureus* isolates, including RN4220 Δ*tarS* and RN4220 Δ*tarM* by incubation with lysostaphin and achromopeptidase (both at 100 μg/mL, Sigma) in 0.5 M NaCl, 10 mM Tris-HCl pH 8.0 at 37°C for 30 min. Samples were boiled for 5 min at 100°C, diluted 5-fold in 1 mM EDTA, 10 mM Tris-HCl pH 8.0 and stored at −20°C until further analysis. The presence of *tarS* and *tarM* was determined by PCR analysis using the following primers: *tarS* (up) 5′- GTGAACATATGAGTAGTGCGTA-3′ and *tarS* (dn) 5′-CATAATGTCCTTCGCCAATCAT-3′ and *tarM* (up) 5′-GGGATACCCATATATTTCAAGG-3′ and *tarM* (dn) 5′- CAATTCGCTTCGTTGGTACCATTC-3’.

To analyze the correlation between the *tar* genotype and expressed WTA glycoprofile on the *S. aureus* surface, bacteria were stained with Fab fragments (10 μg/mL) specific for α-GlcNAc WTA (clone 4461), β-GlcNAc WTA (clone 4497) as described previously,^33^ followed by staining with goat F(ab’)_2_ anti-human kappa-Alexa Fluor 647 (5 μg/mL, Southern Biotech), fixation in PBS 1% formaldehyde and analysis by flow cytometry (BD FACSVerse) using a standardized template. We measured the geometric mean fluorescence intensity (geoMFI) of 10,000 events within a set gate (based on FSC-A/SSC-A).

#### Antibody levels in human plasma (ELISA)

To determine total IgG and IgM levels in human EDTA-plasma samples, Maxisorp plates (Nunc) were coated overnight at 4°C with sheep anti-IgG or sheep anti-IgM (2 μg/mL in PBS, ICN Biomedicals). The next day, the plates were washed three times with PBS 0.05% Tween 20 (PBS-T), blocked for 1 h at 37°C with PBS-T containing 4% BSA (Serva). After three washing cycles with PBS-T, plates were incubated for 1 h at 37°C with a concentration range of plasma samples in duplicate (5-fold serial dilution starting at 1:10,000 for IgG, 1:1,000 for IgM) as well as a standard for either IgG (0.56–200 ng/mL, ChromPure human IgG, Jackson Immunoresearch) or IgM (3.12–400 ng/mL, ChromPure human IgM, Jackson Immunoresearch). Following three washing steps, horseradish peroxidase (HRP)-conjugated goat anti-human IgG or goat anti-human IgM (1:6,000, Southern Biotech) was added for 1 h at 37°C, and after washing the plates were developed using tetramethylbenzidine (TMB). After 5–10 min, the reaction was stopped by adding 1N H_2_SO_4_, absorbance was measured at 450 nm in an iMark Microplate Absorbance Reader (Bio-Rad), and values were corrected for background signals at 595 nm. Pooled human plasma (*n* = 9 healthy donors) was included in every measurement as control sample to determine the inter-assay variation, resulting in a coefficient of variation (CV) < 25% (data not shown).

To determine CHIPS-specific IgM responses in human EDTA-plasma samples, plates were coated overnight at 4°C with recombinant CHIPS (3 μg/mL in PBS), expressed as described previously.^31^ After washing and blocking, similar as described above, plates were incubated for 1 h at 37°C with human EDTA-plasma samples in triplicate at a 1:100 dilution. Heat-inactivated pooled human serum (from *n* = 30 healthy donors, 1:100 dilution) was included as an internal control. After three washing cycles, horseradish peroxidase (HRP)-conjugated goat anti-human IgM (1:2,000, Southern Biotech) was added for 1 h at 37°C, and after washing the plates were developed using TMB substrate (Sigma). The reaction was stopped by adding 1N H_2_SO_4_, and the absorbance was measured at 450 nm on a Synergy H1 Microplate Reader (BioTek). Values were corrected for background signals at 575 nm and normalized using the pooled human serum control, which was set at 1.

### Quantification and statistical analysis

Data obtained by flow cytometry was analyzed using FlowJo 10 (FlowJo LLC). All statistical analyses were performed with Prism software (version 10; GraphPad). Data was checked for normality using the Shapiro-Wilk test, and all statistical details can be found in the figure legends. Data are presented as mean ± standard deviation (SD), unless otherwise stated. Correlations between antibody binding to different WTA beads or *S. aureus* bacteria were assessed using the Spearman correlation, and r values ≥0.6 were considered high correlations. Two-sided *p* values <0.05 were considered significant, and are depicted in the figures.

## Supplemental information

Document S1. Figures S1–S6 and Table S1

Document S2. Article plus supplemental information
