# Supplementary material for: Glycan-specific IgM is critical for human immunity to Staphylococcus aureus
Source: Cell Rep Med. 2024 Sep 17;5(9):101734. doi: 10.1016/j.xcrm.2024.101734 (PMC11525025; doi:10.1016/j.xcrm.2024.101734)
Supplement: Document S1. Figures S1–S6 and Table S1 [file mmc1.pdf]

Cell Reports Medicine, Volume *5*

Supplemental information

Glycan-specific IgM is critical for human immunity to *Staphylococcus aureus*

Astrid Hendriks, Priscilla F. Kerkman, Meri R.J. Varkila, Jelle L.G. Haitsma Mulier, Sara Ali, Thijs ten Doesschate, Thomas W. van der Vaart, Carla J.C. de Haas, Piet C. Aerts, Olaf L. Cremer, Marc J.M. Bonten, Victor Nizet, George Y. Liu, Jeroen D.C. Codée, Suzan H.M. Rooijakkers, Jos A.G. van Strijp, and Nina M. van Sorge

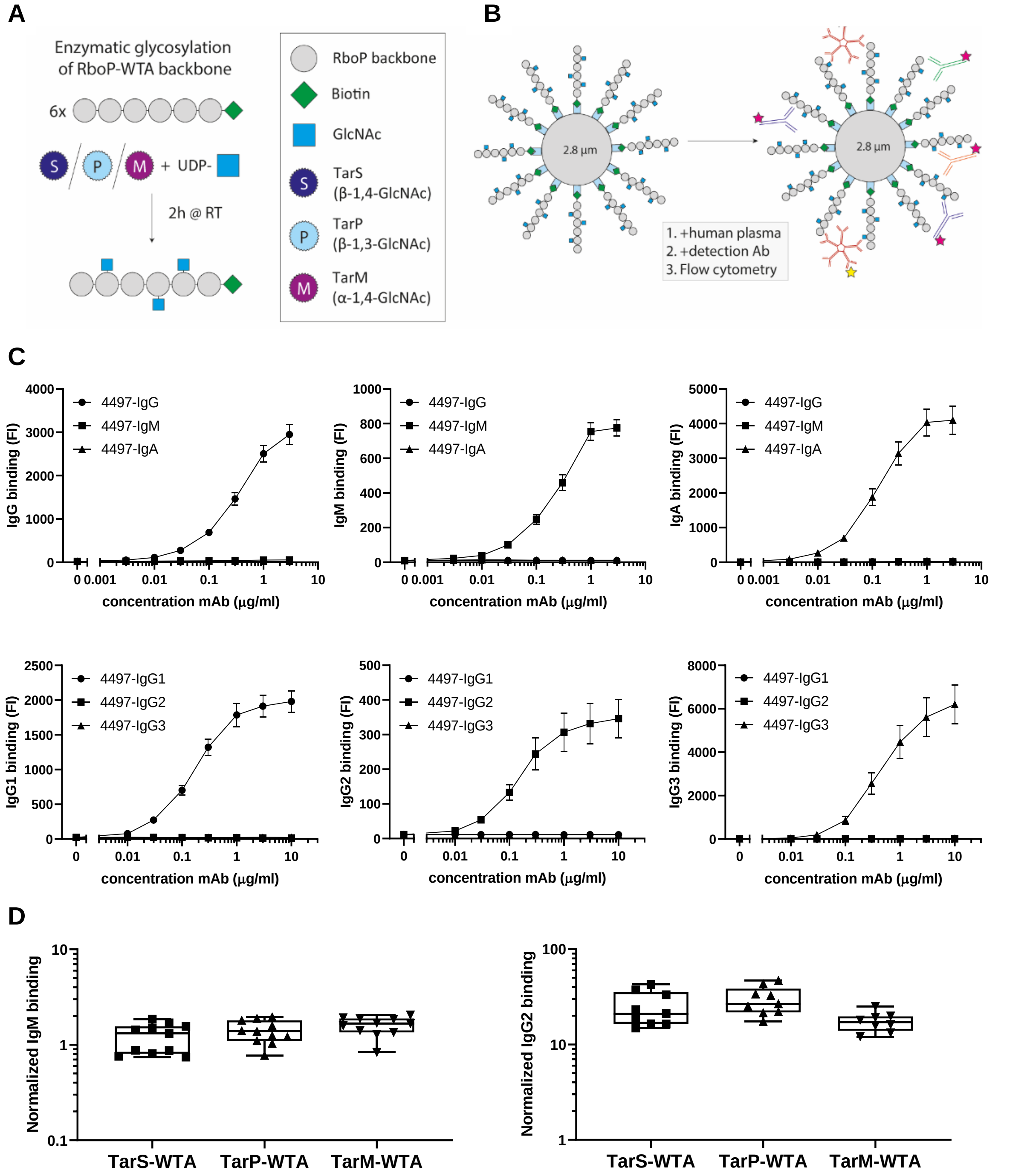

Figure S1. Schematic overview of semi-synthetic WTA fragments, multiplex set up of bead-based assay and interassay variation. Related to STAR Methods. A) Schematic representation of in vitro enzymatic glycosylation of biotinylated RboP-WTA hexamers by recombinant enzymes (TarS, TarP and TarM), which are then coated to beads (WTA beads). B) General work-flow to measure antibody binding to WTA beads by flow cytometry. WTA beads are incubated with human plasma (step 1), subsequently stained with a combination of three detection antibodies to analyze multiple antibody isotypes/subclasses in a single sample (Step 2) to measure antibody binding by flow cytometry (step 3). C) Binding of β-GlcNAc WTA specific mAb (clone 4497 in different Ig isotypes/IgG subclasses) to TarS-WTA coated beads. Concentration curves were used for data interpolation, data is depicted as geometric mean fluorescence intensity (FI) + standard error for the mean (SEM) of 6 (for IgG subclasses) or 11 (for IgM, IgA and IgG) independent experiments. D) Interassay variation within control sample (pooled plasma, n=9 healthy donors) in normalized IgM and IgG2 binding to TarS-WTA, TarP-WTA and TarM-WTA beads.

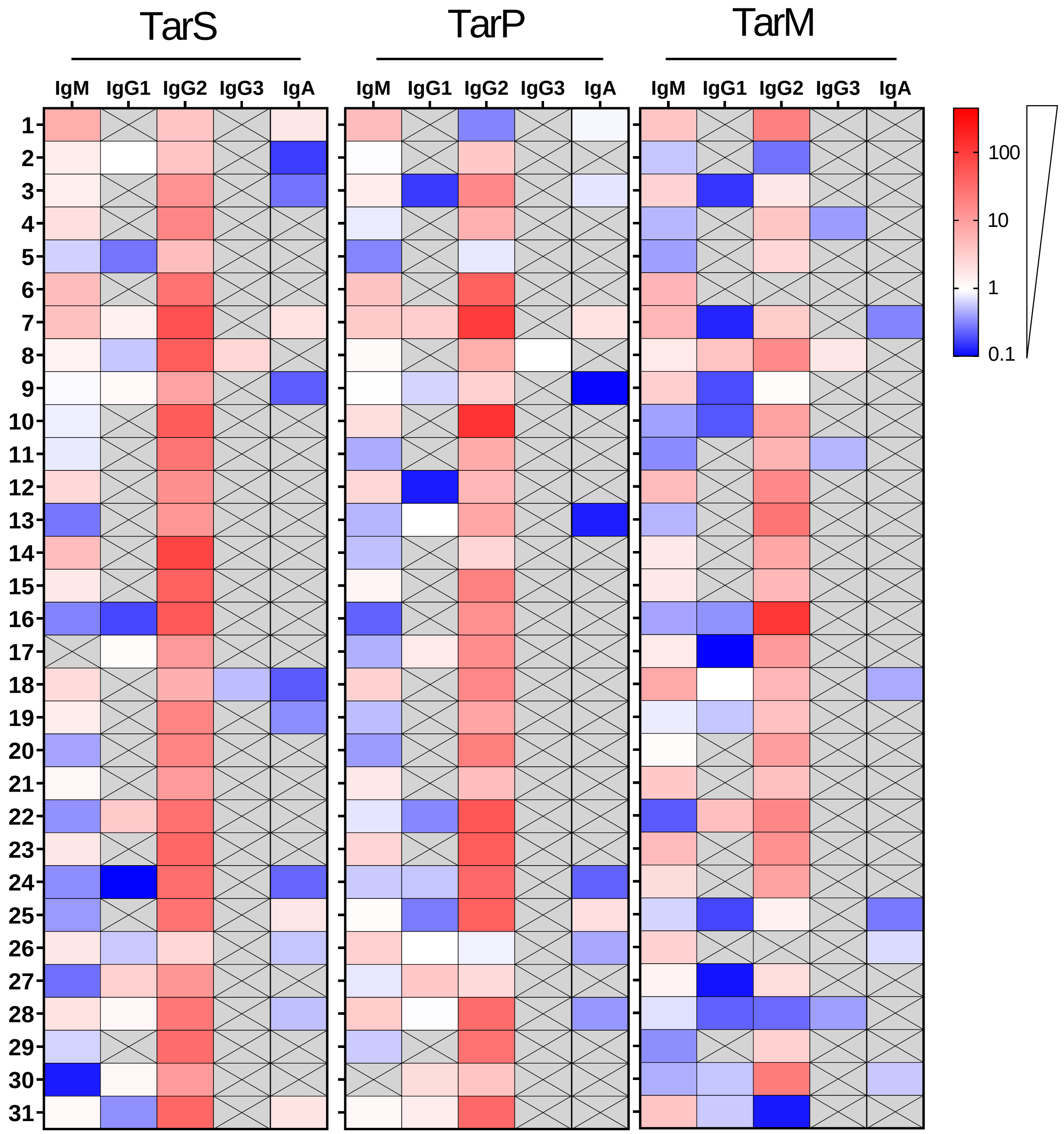

Figure S2. Heatmap of WTA-specific antibody responses in healthy individuals. Related to Figure 1. Colors match log-transformed normalized binding data for IgM, IgG1, IgG2, IgG3 and IgA to the three WTA glycotypes (mediated by TarS, TarP and TarM). Grey boxes with crosses indicate values equal to or below detection limit (0.1).

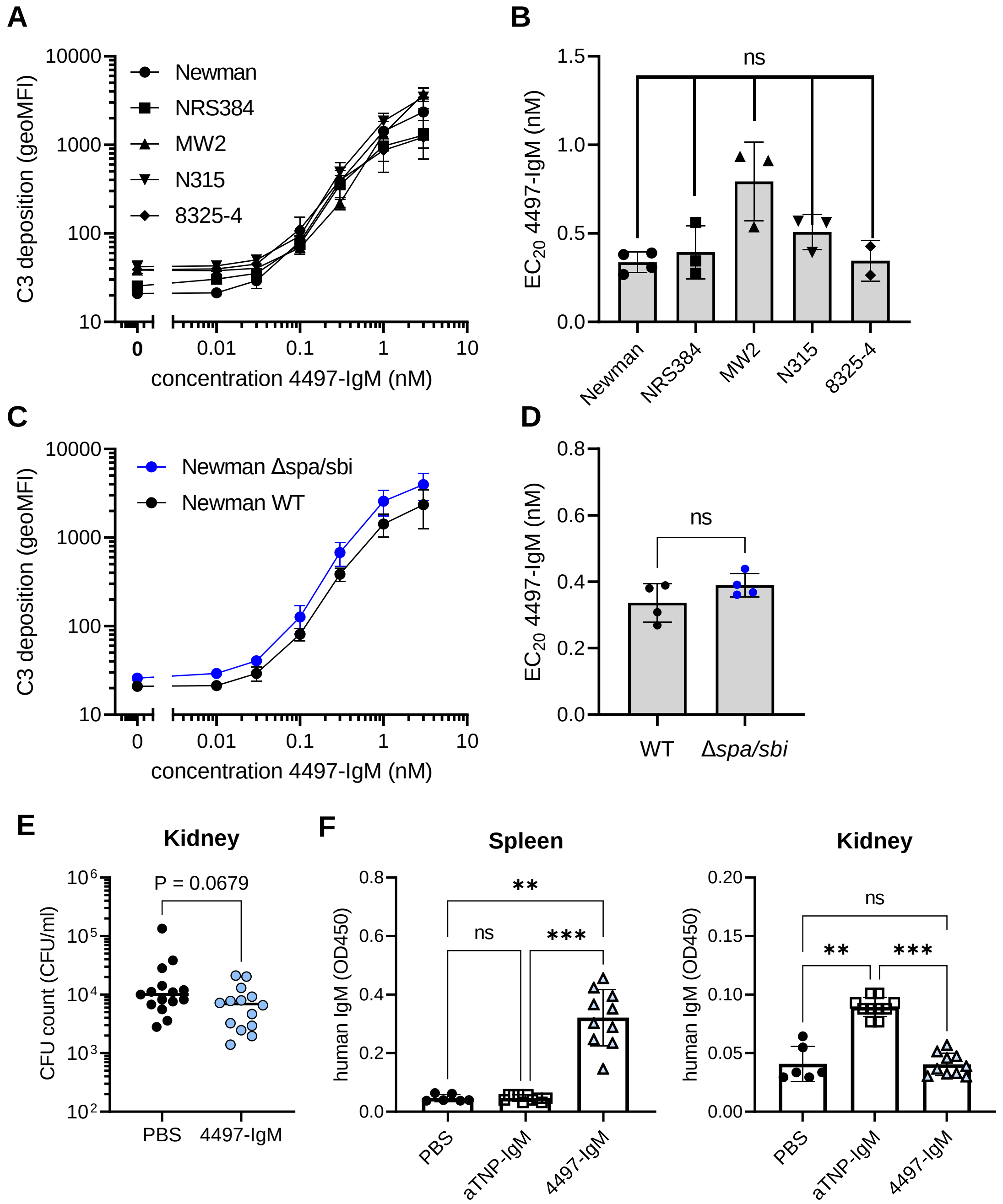

Figure S3. WTA-specific IgM supports immune-mediated clearance of *S. aureus* in vitro and in mouse infection experiments. Related to Figure 2. A) 4497-IgM mediated C3 deposition on *S. aureus* WT strains and (B) related EC20 values. Statistical analysis was performed using a Kruskal-Wallis test with Dunn's correction. C) 4497-IgM mediated C3 deposition and (D) related EC20 values on Newman WT versus protein A (*spa*)-*sbi* deficient *S. aureus* Newman strain. Statistical analysis was performed using a Mann-Whitney test. E) CFU counts in mouse kidneys at 24 h post infection with *S. aureus* N315, passively immunized with 30 ug 4497-IgM (n=14) or PBS control (n=15). Statistical analysis was performed using a Mann-Whitney test. F) Presence of passively-administrated human IgM (30 µg anti-TNP IgM, 4497-IgM or PBS control) in homogenized organs (spleen and kidney) from mice 24 h post infection with *S. aureus* (n=6 for PBS control, n=10 for anti-TNP-IgM and 4497-IgM). The three groups were compared for statistical analysis using a Kruskal-Wallis test with Dunn's correction.

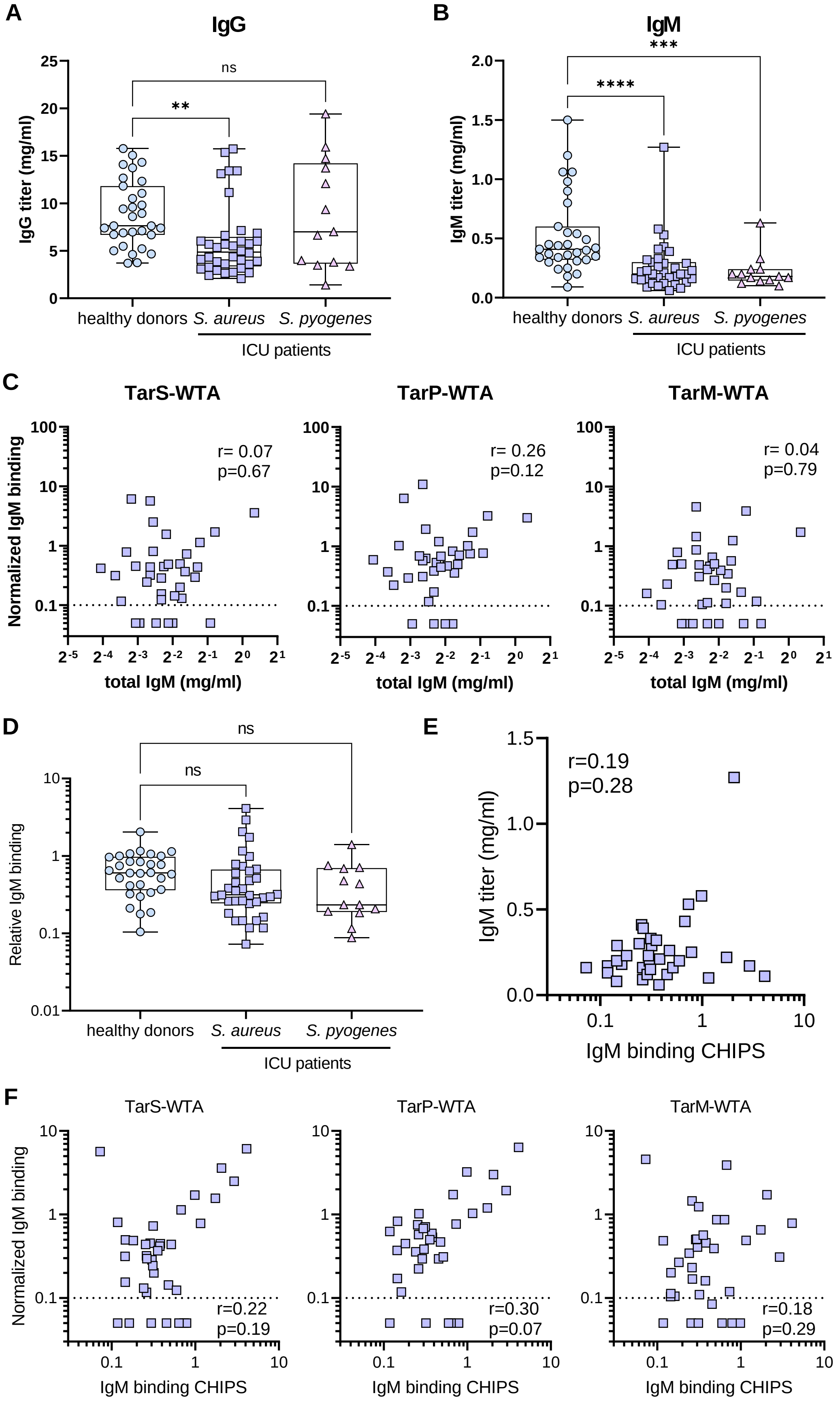
A
IgG
IgG titer (mg/ml)
ns
**
healthy donors
S. aureus
S. pyogenes
ICU patients
B
IgM
IgM titer (mg/ml)
***
****
healthy donors
S. aureus
S. pyogenes
ICU patients
C
TarS-WTA
TarP-WTA
TarM-WTA
Normalized IgM binding
r= 0.07
p=0.67
r= 0.26
p=0.12
r= 0.04
p=0.79
total IgM (mg/ml)
total IgM (mg/ml)
total IgM (mg/ml)
D
ns
ns
Relative IgM binding
healthy donors
S. aureus
S. pyogenes
ICU patients
E
r=0.19
p=0.28
IgM titer (mg/ml)
IgM binding CHIPS
F
TarS-WTA
TarP-WTA
TarM-WTA
Normalized IgM binding
r=0.22
p=0.19
r=0.30
p=0.07
r=0.18
p=0.29
IgM binding CHIPS
IgM binding CHIPS
IgM binding CHIPS

Figure S4. Specificity of WTA-specific IgM antibody levels in healthy donors and bacteremia patients. Related to Figure 3. A) IgG titers and B) IgM titers of healthy donors (n=31) and patients with *S. aureus* (n=36) or *S. pyogenes* (n=13) bacteremia. C) Spearman correlation between WTA-specific IgM responses and total IgM titers in patients with *S. aureus* bacteremia. D) CHIPS-IgM responses in healthy donors (n=31) and patients with *S. aureus* (n=36) or *S. pyogenes* (n=13) bacteremia. dotted line represents detection limit. Symbols indicate individual donors, boxplots extend from the 25th to 75th percentiles and the line inside the box represents the median. E) Spearman correlation between CHIPS-specific IgM responses and total IgM titers in patients with *S. aureus* bacteremia. F) Spearman correlation between CHIPS- and WTA-specific IgM responses in patients with *S. aureus* bacteremia. ***$p < 0.001$, ****$p < 0.0001$, ns=not significant.

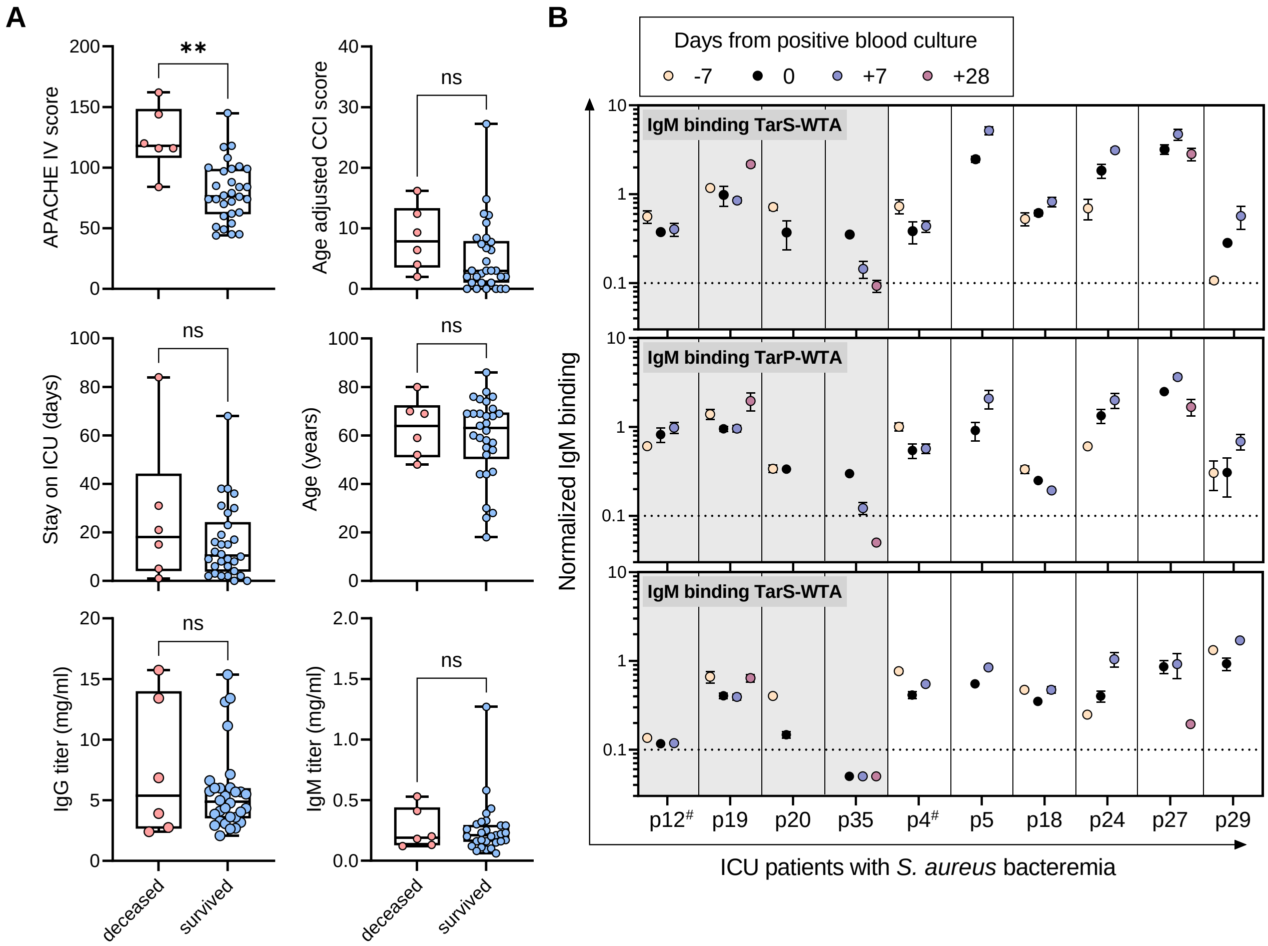

Figure S5. Clinical data and longitudinal analysis of WTA-specific IgM responses of ICU patients with *S. aureus* bacteremia. Related to Figure 4. A) Clinical data of patients grouped on ICU mortality, including APACHE IV score, age-adjusted Charlson Comorbidity Index (CCI), length of ICU stay, age, IgG and IgM titers. Symbols indicate individual donors, boxplots extend from the 25th to 75th percentiles and the line inside the box represents the median. Statistical analysis was performed using Mann-Whitney test. B) Normalized IgM binding for TarS-WTA, TarP-WTA and TarM-WTA at different time points (in days from positive blood culture) in selected patients. Dotted line represents the lower limit of detection, symbols shown below line present extrapolated values.

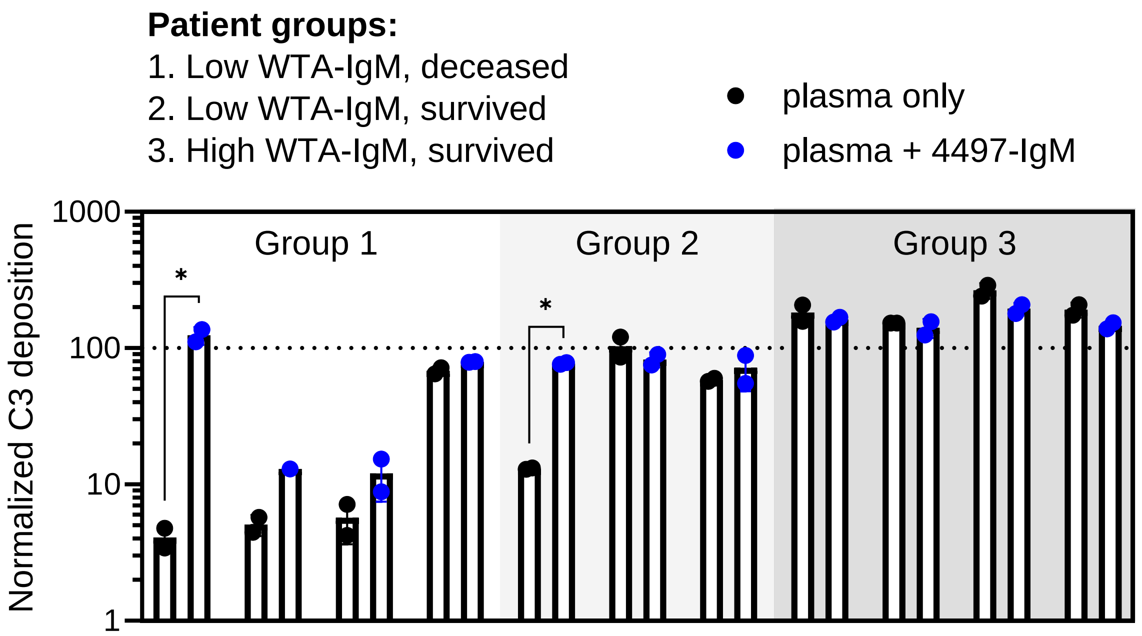

Figure S6. Effect of 4497-IgM mAb on antibody-mediated complement deposition on *S. aureus* in presence of patient plasma. Related to Figure 5. Normalized C3 deposition on *S. aureus* NRS384 WT or Δ*tarM*, dependent on WTA glycoprofile of clinical *S. aureus* isolate, pre-opsonized with patient plasma (1:50 dilution) with or without 4497-IgM (10 nM). Patients were stratified in three groups, based on WTA-IgM titers and ICU mortality. Data was normalized using C3 deposition by 4497-IgM only (set at 100%, dotted line). Statistical analysis was performed using multiple unpaired t-tests. *p < 0.05

Table S1. Protein sequence for 4497 antibody isotypes. Related to STAR Methods.

	**Sequence**
**Variable heavy chain**	
Anti-WTA-IgG (4497)	EVQLVESGGGLVQPGGSLRLSCSASGFSFNSFWMHWVRQVPGKGLVWI SFTNNEGTTTAYADSVRGRFIISRDNAKNTLYLEMNNLRGEDTAVYYCAR GDGGLDDWGQGTLVTVSS
**Variable light chain**	
Anti-WTA-IgG (4497)	DIQLTQSPDSLAVSLGERATINCKSSQSIFRTSRNKNLLNWYQQRPGQPP RLLIHWASTRKSGVPDRFSGSGFGTDFTLTITSLQAEDVAIYYCQQYFSPP YTFGQGTKLEIK
**Constant heavy chain**	
IgG1	ASTKGPSVFPLAPSSKSTSGGTAALGCLVKDYFPEPVTVSWNSGALTSG VHTFPAVLQSSGLYSLSSVVTVPSSSLGTQTYICNVNHKPSNTKVDKKVE PKSCDKTHTCPPCPAPELLGGPSVFLFPPKPKDTLMISRTPEVTCVVVDV SHEDPEVKFNWYVDGVEVHNAKTKPREEQYNSTYRVVSVLTVLHQDWL NGKEYKCKVSNKALPAPIEKTISKAKGQPREPQVYTLPPSREEMTKNQVS LTCLVKGFYPSDIAVEWESNGQPENNYKTTPPVLDSDGSFFLYSKLTVDK SRWQQGNVFSCSVMHEALHNHYTQKSLSLSPGK
IgG2	ASTKGPSVFPLAPCSRSTSESTAALGCLVKDYFPEPVTVSWNSGALTSG VHTFPAVLQSSGLYSLSSVVTVPSSNFGTQTYTCNVDHKPSNTKVDKTVE RKCCVECPPCPAPPVAGPSVFLFPPKPKDTLMISRTPEVTCVVVDVSHED PEVQFNWYVDGVEVHNAKTKPREEQFNSTFRVVSVLTVVHQDWLNGKE YKCKVSNKGLPAPIEKTISKTKGQPREPQVYTLPPSREEMTKNQVSLTCL VKGFYPSDIAVEWESNGQPENNYKTTPPMLDSDGSFFLYSKLTVDKSRW QQGNVFSCSVMHEALHNHYTQKSLSLSPGK
IgG3	ASTKGPSVFPLAPCSRSTSGGTAALGCLVKDYFPEPVTVSWNSGALTSG VHTFPAVLQSSGLYSLSSVVTVPSSSLGTQTYTCNVNHKPSNTKVDKRVE LKTPLGDTTHTCPRCPEPKSCDTPPPCPRCPEPKSCDTPPPCPRCPEPK SCDTPPPCPRCPAPELLGGPSVFLFPPKPKDTLMISRTPEVTCVVVDVSH EDPEVQFKWYVDGVEVHNAKTKPREEQYNSTFRVVSVLTVLHQDWLNG KEYKCKVSNKALPAPIEKTISKTKGQPREPQVYTLPPSREEMTKNQVSLT CLVKGFYPSDIAVEWESSGQPENNYNTTPPMLDSDGSFFLYSKLTVDKS RWQQGNIFSCSVMHEALHNRFTQKSLSLSPGK
IgM	GSASAPTLFPLVSCENSPSDTSSVAVGCLAQDFLPDSITFSWKYKNNSDI SSTRGFPSVLRGGKYAATSQVLLPSKDVMQGTDEHVVCKVQHPNGNKE KNVPLPVIAELPPKVSVFVPPRDGFFGNPRKSKLICQATGFSPRQIQVSWL REGKQVGSGVTTDQVQAEAKESGPTTYKVTSTLTIKESDWLSQSMFTCR VDHRGLTFQQNASSMCVPDQDTAIRVFAIPPSFASIFLTKSTKLTCLVTDL TTYDSVTISWTRQNGEAVKTHTNISESHPNATFSAVGEASICEDDWNSGE RFTCTVTHTDLPSPLKQTISRPKGVALHRPDVYLLPPAREQLNLRESATIT CLVTGFSPADVFVQWMQRGQPLSPEKYVTSAPMPEPQAPGRYFAHSILT VSEEEWNTGETYTCVVAHEALPNRVTERTVDKSTGKPTLYNVSLVMSDT AGTCY
IgA1	ASPTSPKVFPLSLCSTQPDGNVVIACLVQGFFPQEPLSVTWSESGQGVT ARNFPPSQDASGDLYTTSSQLTLPATQCLAGKSVTCHVKHYTNPSQDVT VPCPVPSTPPTPSPSTPPTPSPSCCHPRLSLHRPALEDLLLGSEANLTCTL TGLRDASGVTFTWTPSSGKSAVQGPPERDLCGCYSVSSVLPGCAEPWN HGKTFTCTAAYPESKTPLTATLSKSGNTFRPEVHLLPPPSEELALNELVTL TCLARGFSPKDVLVRWLQGSQELPREKYLTWASRQEPSQGTTTFAVTSI LRVAAEDWKKGDTFSCMVGHEALPLAFTQKTIDRLAGKPTHVNVSVVMA EVDGTCY
**Constant light chain (kappa)**	
IgG1,2,3, IgM, IgA1	RTVAAPSVFIFPPSDEQLKSGTASVVCLLNNFYPREAKVQWKVDNALQS GNSQESVTEQDSKDSTYSLSSTLTLSKADYEKHKVYACEVTHQGLSSPV TKSFNRGEC
